# Ninein is essential for apico-basal microtubule formation and CLIP-170 facilitates its redeployment to non-centrosomal microtubule organizing centres

**DOI:** 10.1098/rsob.160274

**Published:** 2017-02-08

**Authors:** Deborah A. Goldspink, Chris Rookyard, Benjamin J. Tyrrell, Jonathan Gadsby, James Perkins, Elizabeth K. Lund, Niels Galjart, Paul Thomas, Tom Wileman, Mette M. Mogensen

**Affiliations:** 1 School of Biological Sciences, University of East Anglia, Norwich, UK; 2 School of Computing Science, University of East Anglia, Norwich, UK; 3 Medical School, University of East Anglia, Norwich, UK; 4 Department of Cell Biology and Genetics, Erasmus MC, Rotterdam, The Netherlands

**Keywords:** microtubules, ninein, CLIP-170, IQGAP1, Rac1, non-centrosomal MTOCs

## Abstract

Differentiation of columnar epithelial cells involves a dramatic reorganization of the microtubules (MTs) and centrosomal components into an apico-basal array no longer anchored at the centrosome. Instead, the minus-ends of the MTs become anchored at apical non-centrosomal microtubule organizing centres (n-MTOCs). Formation of n-MTOCs is critical as they determine the spatial organization of MTs, which in turn influences cell shape and function. However, how they are formed is poorly understood. We have previously shown that the centrosomal anchoring protein ninein is released from the centrosome, moves in a microtubule-dependent manner and accumulates at n-MTOCs during epithelial differentiation. Here, we report using depletion and knockout (KO) approaches that ninein expression is essential for apico-basal array formation and epithelial elongation and that CLIP-170 is required for its redeployment to n-MTOCs. Functional inhibition also revealed that IQGAP1 and active Rac1 coordinate with CLIP-170 to facilitate microtubule plus-end cortical targeting and ninein redeployment. Intestinal tissue and *in vitro* organoids from the *Clip1/Clip2* double KO mouse with deletions in the genes encoding CLIP-170 and CLIP-115, respectively, confirmed requirement of CLIP-170 for ninein recruitment to n-MTOCs, with possible compensation by other anchoring factors such as p150^Glued^ and CAMSAP2 ensuring apico-basal microtubule formation despite loss of ninein at n-MTOCs.

## 1. Introduction

Apico-basal polarization and differentiation of simple epithelial cells such as those of the kidney and intestine involve a dramatic reorganization not only of the microtubules (MTs) but also of centrosomal components. The radial MT array focused on a centrally located centrosomal microtubule organizing centre (MTOC) found in undifferentiated epithelial cells reorganizes during differentiation to form a mainly non-centrosomal apico-basal array with minus-ends directed apically [1–5]. In polarized epithelia such as kidney, the minus-ends of the apico-basal MTs become anchored at apical non-centrosomal MTOCs (n-MTOCs) associated with adherens junctions (AJ, zonula adherens) [6]. Centrosomal anchoring proteins including ninein relocate to these sites, colocalizing with β-catenin and the dynactin component p150^Glued^ [6–8]. Analyses of MT reorganization based on regrowth following nocodazole removal and live GFP-EB1 imaging in kidney (MDCK) cells have established that the apico-basal MTs originate from the centrosome, but the vast majority subsequently become anchored at apical n-MTOCs [6,9,10]. However, in terminally differentiated intestinal epithelial cells, both nucleating and anchoring components are redeployed to apical surface-associated n-MTOCs [11,12]. A n-MTOC may thus act either as an anchoring site or as a nucleating and anchoring site for non-centrosomal MTs. n-MTOCs are critical as they determine the temporal and spatial MT anchorage and organization, which in turn influences the shape and function of epithelial cells. However, the mechanisms responsible for MT minus-end anchorage and formation of n-MTOCs are poorly understood.

The importance of the centrosomal protein ninein in development is evident through studies showing that it influences neurogenesis, angiogenesis and stem cell fate, and that *Nin* gene mutations cause human disorders such as microcephalic primordial dwarfism and spondyloepimetaphyseal dysplasia [13–17]. Ninein is a large coiled-coil protein that associates with the subdistal appendages of the mother centriole and the minus-ends of both centrioles [7]. Loss- and gain-of-function studies have established that ninein acts as a major MT minus-end anchor at the centrosome, but whether this is also the case at n-MTOCs in polarized epithelial cells remains to be established [18,19]. Analyses of *in situ* inner ear epithelial cells revealed that ninein gradually relocates to apical non-centrosomal anchoring sites during inner ear morphogenesis, while live-cell imaging showed that GFP-ninein speckles move to and from the centrosome in a MT-dependent manner [7,8,20]. Relocation of ninein from the centrosome to cortical sites has also been reported during epidermis differentiation [21]. However, the molecular mechanisms responsible for the relocation of ninein during polarized epithelial differentiation still remain to be determined.

MT plus-end tracking proteins (+TIPs) have proved essential for MT reorganization during differentiation of epithelia and skeletal muscle [22–24]. CLIP-170 was the first +TIP characterized [25] and was shown to accumulate at MT plus-ends and act as a rescue factor [26]. CLIP-170, CLIP-115 and p150^Glued^ bind MTs and EB1 through CAP-Gly domains [27]. MT plus-end cortical interactions facilitated by +TIPs have proved important for several cellular processes such as directed cell migration, centrosome repositioning, spindle orientation and adherens and gap junction formation. For example, EB1, dynein/dynactin and CLIP-170 mediate MT cortical capture at the leading edge of migrating cells and at AJs, with CLIP-170 shown to target AJs prior to apico-basal array assembly [6,28–30]. MT plus-end cortical interactions and CLIP-170 may thus facilitate delivery of ninein to n-MTOCs and promote the formation of non-centrosomal apico-basal MT arrays in differentiating epithelial cells. The main focus of this investigation was, therefore, to determine whether CLIP-170 is required for redeployment of ninein to n-MTOCs during epithelial differentiation. Additionally, the involvement of active Rac1 and the cortical receptor IQGAP1 was also explored, as these two proteins have been shown to interact with CLIP-170, form a complex and capture MT plus-ends at the cortex [31].

Here, we show that ninein expression is essential for apico-basal MT formation and columnar epithelial shape. We also show that ninein and CLIP-170 localize to apical junction-associated n-MTOCs in fully differentiated MDCKII cysts and apical surface n-MTOCs in terminally differentiated (villus) epithelial cells of *ex vivo* intestine and *in vitro* organoids generated from mouse small intestine. We also identify p150^Glued^, γ-tubulin and calmodulin-regulated spectrin-associated protein 2 (CAMSAP2) at the n-MTOCs in villus tissue and organoids. Using *in vitro* and *ex vivo* depletion and knockout (KO) studies, we show that CLIP-170, IQGAP1 and active Rac1 influence MT plus-ends cortical contact and facilitate redeployment of ninein to apical n-MTOCs. We propose a model for ninein redeployment in which CLIP-170-bound MT plus-ends target and are captured by IQGAP1 cortical receptors in a process promoted by active Rac1. In addition, the *Clip1/Clip2* double KO mouse with deletions in the genes encoding CLIP-170 and CLIP-115, respectively, confirmed the requirement of CLIP-170 for ninein recruitment to n-MTOCs and suggests engagement of a compensation mechanism to ensure non-centrosomal apico-basal MT formation in the absence of CLIP-170 and ninein at n-MTOCs.

## 2. Results

### 2.1. Ninein siRNA depletion inhibits apico-basal microtubule bundle formation and epithelial cell elongation

**Figure 1.**
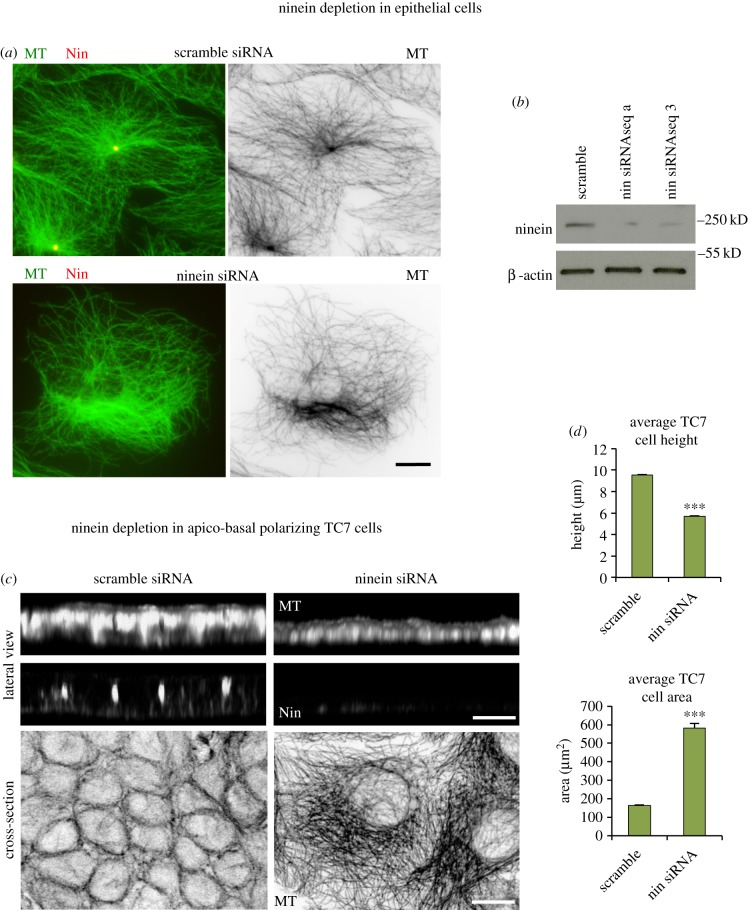
Ninein depletion in epithelial cells. (*a*) Scramble and ninein siRNA (seq a) depleted TC7 cells methanol fixed and stained for MTs (mAb YL1/2; green; invert) and ninein (mAb N5; red), showing loss of radial MT organization and centrosomal focus in depleted cell. (*b*) Western blot of cell lysates of scramble and ninein (seq a and 3) siRNA showing ninein (mAb Bethyl) and β-actin expression. (*c*) Confocal optical sections and three-dimensional reconstructions of scramble and ninein siRNA (seq a) depleted TC7 cells seeded for apico-basal MT array formation, fixed in methanol and labelled for MTs (mAb YL1/2) and ninein (pAb Pep3). (*d*) Analysis of cell height (scramble *n* = 284, nin siRNA seq a = 251) and cross-sectional area (scramble *n* = 190, nin siRNA seq a = 200) in scramble and ninein siRNA-treated TC7 cells show decreased cell height and increased area in depleted cell (Mann–Whitney *U*-test, ***p < 0.001). Scale bars, 10 µm.

Although ninein is needed for centrosomal MT anchorage, its role in apico-basal MT array formation is not known. Human TC7 colonic cells, which readily elongate and produce 10–12 µm tall cells with apico-basal arrays when grown to confluence, were used as a model to investigate the role of ninein in apico-basal MT array formation [22]. Ninein siRNA depletion was performed using previously tested sequences [8,15], which as expected [19] produced loss of centrosomal anchorage and disorganized MTs in non-confluent epithelial cells (figure 1*a*,*b*).

In confluent TC7 cells, a typical apico-basal epithelial MT organization was evident in scramble siRNA cells, with lateral views showing apico-basal alignment of MTs and cross sections revealing peripheral MT rings representing optical cross sections of the apico-basal array (figure 1*c*). However, ninein knockdown revealed a striking lack of cell elongation and apico-basal MTs with optical cross sections through the middle region, instead showing disorganized networks within threefold larger cells (figure 1*c*,*d*). These findings show that ninein expression is critical for apico-basal MT array formation and epithelial cell elongation.

### 2.2. Ninein and CLIP-170 localize to apical n-MTOCs in three-dimensional MDCKII cysts and are part of the membrane fraction

**Figure 2.**
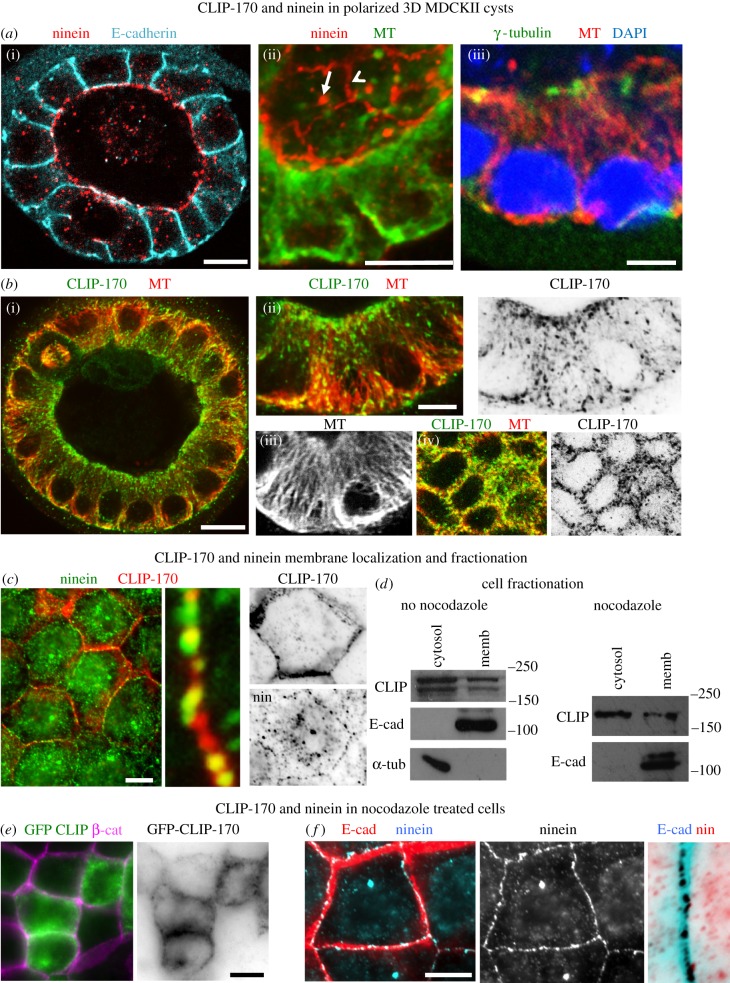
CLIP-170 and ninein in confluent and fully differentiated MDCKII cysts. (*a*,*b*) Cells grown in Matrigel to form three-dimensional cysts. (*a*) Optical sections of cysts fixed in methanol and stained for ninein (pAb Pep3; red) and E-cadherin (mAb, blue) showing apical localization in (*a*(i)) and cyst regions showing apico-basal MTs (mAb YL1/2, green in (*a*(ii)); pAb α-tubulin, red in (*a*(iii))). Optical oblique section though cyst region in (*a*(ii)) shows both apical and baso-lateral views with ninein (pAb N5; red) at apical cortex (arrowhead) and centrosomes (arrow) in polarized epithelial cells. Baso-lateral view of cyst epithelial cells in (*a*(iii)) shows γ-tubulin (green, (*a*(iii))) at centrosomes. (*b*) Optical section of cyst fixed in formaldehyde–methanol and stained for CLIP-170 (pAb, green) and MTs (mAb YL1/2, red) (*b*(i)) and cyst regions revealing apico-basal MTs (red; (*b*(ii));(*b*(iii))) and apical concentration of CLIP-170 colocalizing with MTs at apical cortex (*b*(iv)). (*c*) Confluent cells fixed in methanol and labelled for ninein (pAb Pep3, green) and CLIP-170 (mAb F-3, red) showing some colocalization (yellow) at cortical regions. (*d*) Western blots of fractionated control and nocodazole-treated cell lysates showing cytosol and membrane fractions, blots probed for CLIP-170 (pAb), E-cadherin (mAb) and α-tubulin (pAb). Note that the double band for CLIP-170 is absent in the nocodazole-treated cell extract and this is most likely due to nocodazole-induced dephosphorylation [33]. (*e*) Nocodazole-treated cells expressing GFP-CLIP-170 (green) fixed in methanol and labelled for β-catenin (pAb, purple) showing cortical rings of GFP-CLIP-170. (*f*) Nocodazole-treated cells fixed in methanol and stained for ninein (pAb Pep3, blue) and E-cadherin (mAb, red). Enlarged inverted junctional region showing cortical ninein remains at the cell cortex following nocodazole treatment. Scale bars, 10 µm except for (*a*(iii)) and (*b*(ii)), 5 µm.

Cortical ninein and CLIP-170 have previously been identified and localized in two-dimensional *in vitro* confluent and polarized MDCKII cell layers, and this cell model was therefore used for further analysis of junction-associated n-MTOCs [6,8,32]. Here, we show three-dimensional MDCKII cysts grown in Matrigel with differentiated epithelial cells possessing distinct apico-basal MT arrays, apical centrosomes and ninein and CLIP-170 at apical junction-associated n-MTOCs (figure 2*a*,*b*). An apical peripheral ring of ninein and CLIP-170 is evident, which colocalizes with the minus-ends of the apico-basal MTs (figure 2*a*(ii),*b*(iv)). CLIP-170 comets and ninein speckles are also present in the cytoplasm, and ninein is evident at the apical centrosome while γ-tubulin is present at the centrosome but absent from the n-MTOCs (figure 2*a*(ii)(iii),*b*(i)(ii)).

Some co-localization of ninein and CLIP-170 was evident in confluent MDCKII cells; however, coimmunoprecipitation (Co-IP) did not reveal any complex formation between ninein and CLIP-170 (figure 2*c* and data not shown). Expression of GFP-CLIP-170 also revealed accumulation at the cell cortex, and cell fractionation confirmed endogenous CLIP-170 within the membrane and cytosolic fractions (figure 2*d*,*e*). Nocodazole treatment to depolymerize MTs revealed that both endogenous and GFP-CLIP-170 remained at the cortex and that CLIP-170 was still in the membrane fraction (figure 2*d*,*e*). Similarly, cortical ninein remained at the cortex in the presence of nocodazole (figure 2*f*). Interestingly, a proteomics study has also identified ninein and CLIP-170 in the membrane fraction of U20S cells (peptracker.com) [34]. This suggests that both ninein and CLIP-170 are associated with the cortex and that they are bound there independently of MTs.

### 2.3. Differentiated intestinal epithelia and organoids show accumulation of ninein and CLIP-170 at apical surface n-MTOCs

**Figure 3.**
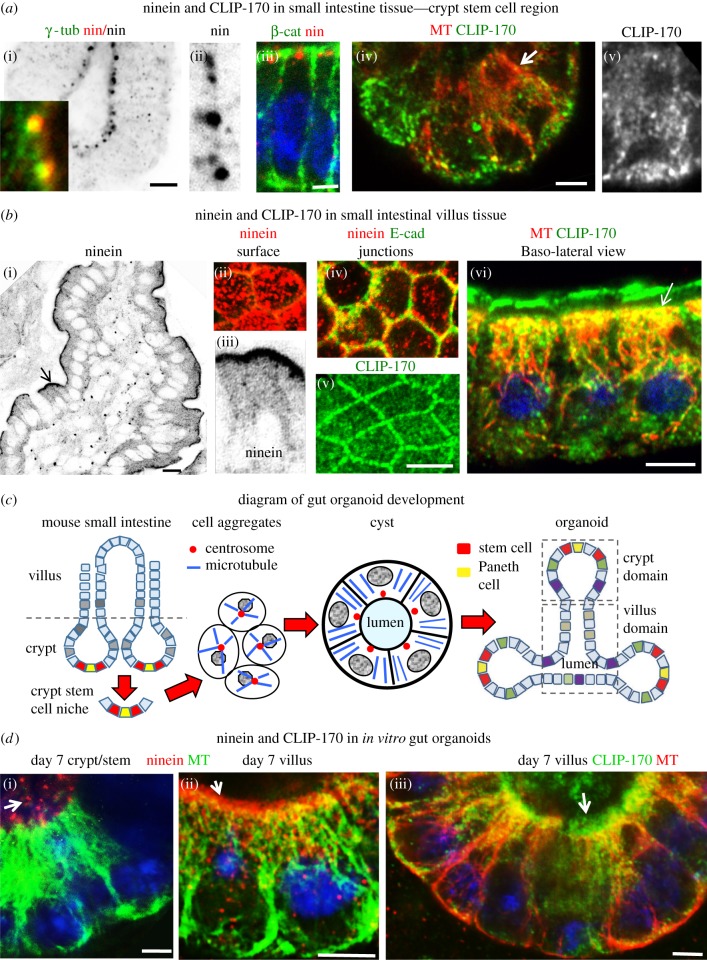
Ninein and CLIP-170 in mouse small intestinal tissue and organoids. (*a*) Isolated basal region of small intestine crypts fixed in methanol (*a*(i–iii)) or formaldehyde–methanol (*a*(iv,v)) and stained for ninein (pAB Pep3, red) (*a*(i–iii)), γ-tubulin (mAb, green in (*a*(i))), β-catenin (mAb, green in (*a*(iii))), CLIP-170 (pAb, green; arrow in (*a*(iv)) enlarged region in (*a*(v))) and MTs (mAb YL1/2, red in (*a*(iv))). Apico-basal MTs are evident in cells of the stem cell region (*a*(iv)) but ninein is concentrated at the apical centrosome ((*a*(i–iii))), where it colocalizes with γ-tubulin (inset in (*a*(i))). CLIP-170 is present as comets in cells within the stem cell region (*a*(iv,v)). (*b*) Confocal images of small intestine villus fixed in methanol (*b*(i–iv)) or formaldehyde–methanol (*b*(v,vi)) and stained for ninein (pAb Pep3, red) and CLIP-170 (pAb, green) localized at n-MTOCs at cell apices. (*b*(i,iii)) Cryostat section of villus with apical ninein localization (invert, arrow enlarged region in (*b*(iii))). (*b*(ii,iv)) Optical sections through whole mount villus showing apical views of apical surface (*b*(ii)) and junctions (*b*(iv)) (E-cadherin, mAb, green) with ninein (pAb Pep3, red) puncta at apical surface and AJ-associated n-MTOCs. (*b*(v,vi)) Optical sections of whole mount villus stained for CLIP-170 (pAb, green) and MTs (mAb YL1/2, red) showing cross-sectional view (*b*(v)) of CLIP-170 at apical junctional n-MTOCs and lateral view (*b*(vi)) of villus cells with CLIP-170 concentrated at apical surface n-MTOCs (arrow) and along length of MTs. (*c*) Diagram showing small intestine with crypt and villus regions and organoid generation from isolated mouse small intestinal stem cells initially leading to the formation of cell aggregates that develop into cysts and then into organoids with crypt and villus domains. (*d*) MTs (mAb YL1/2, green in (*d*(i,ii)) and red in (*d*(iii)), Ninein (pAb Pep3, red) and CLIP-170 (pAb, green) in 7-day cultured gut organoids showing apico-basal MT (mAb YL1/2) arrays in both crypt and villus domains, with ninein concentrated at apical centrosomes (arrow in (*d*(i))) in stem cell region of crypt and ninein (arrow in (*d*(ii)) and CLIP-170 (arrow in (*d*(iii))) at apical surface n-MTOCs in villus-domain cells. Scale bars, 5 µm except for (*b*(i)), 10 µm.

Although the two- and three-dimensional *in vitro* epithelial cultures show that a fraction of CLIP-170 and ninein localize to apical cortical n-MTOCs during differentiation, it is important to determine whether this is the case *in vivo* and if this is linked to differentiation. The intestinal epithelium is a good model to investigate the redeployment of ninein during differentiation as it contains both proliferating and differentiated epithelial cells. A hierarchy of differentiation is evident in the small intestine. Stem cells at the bottom of crypts give rise to immature transit-amplifying cells that proliferate and gradually differentiate as they migrate up the crypt into the villus, where they become fully differentiated enterocytes prior to being shed into the lumen [35] (figure 3*c*). The stem cell niche at the bottom of the crypts thus contains undifferentiated while the upper villus contains terminally differentiated epithelial cells.

In the stem cell region of the crypts, ninein was concentrated at the apical centrosome, where it colocalized with γ-tubulin, while CLIP-170 comets were evident throughout the cytoplasm (figure 3*a*). No discernable accumulation of ninein or CLIP-170 was apparent at apical cortical sites. By contrast, terminally differentiated villus cells, which lack centrosomes and have γ-tubulin at the apical surface [11,36], revealed distinct apical bands of both ninein and CLIP-170 at the apical surface and at junctions (figure 3*b*). Ninein and CLIP-170 were present not only at the AJs (figure 3*b*(iv)(v)) but also just below the apical surface (figure 3*b*(ii)(vi)). CLIP-170 was also present along the lattice of the apico-basal MTs (figure 3*b*(vi)). Fully differentiated epithelial cells of the villus thus exhibited distinct non-centrosomal apico-basal MT bundles with minus-ends anchored within apical n-MTOCs containing ninein and CLIP-170.

*In vitro* gut organoids, also referred to as ‘mini-guts’, are reported to mimic the architecture and morphogenesis of the *in vivo* gut, but whether this includes centrosomal reorganization during differentiation has not been established. Here, we generated gut organoids from mouse small intestine as previously described [37] (figure 3*c*). Cells from the stem cell niche proliferate forming aggregates and cysts that subsequently generate crypt-like buds with stem cells at the base and differentiation gradually progressing towards the cyst region, which becomes villus-like (figure 3*c*; electronic supplementary material, figure S1). Apico-basal MT arrays, which form during gut organoid development, were evident in cells of both the proliferating stem cell niche (base of crypts) and fully differentiated villus domains (figure 3*d*). However, as for the *ex vivo* tissue data, ninein was concentrated at the centrosome in crypt/stem cells (figure 3*d*(i)) while both ninein and CLIP-170 localized at the apical n-MTOC in cells of the organoid villus domains (figure 3*d*(ii)(iii)).

### 2.4. CLIP-170 siRNA knockdown in MDCKII cells reveals marked reduction in cortical ninein and reduced cyst size

**Figure 4.**
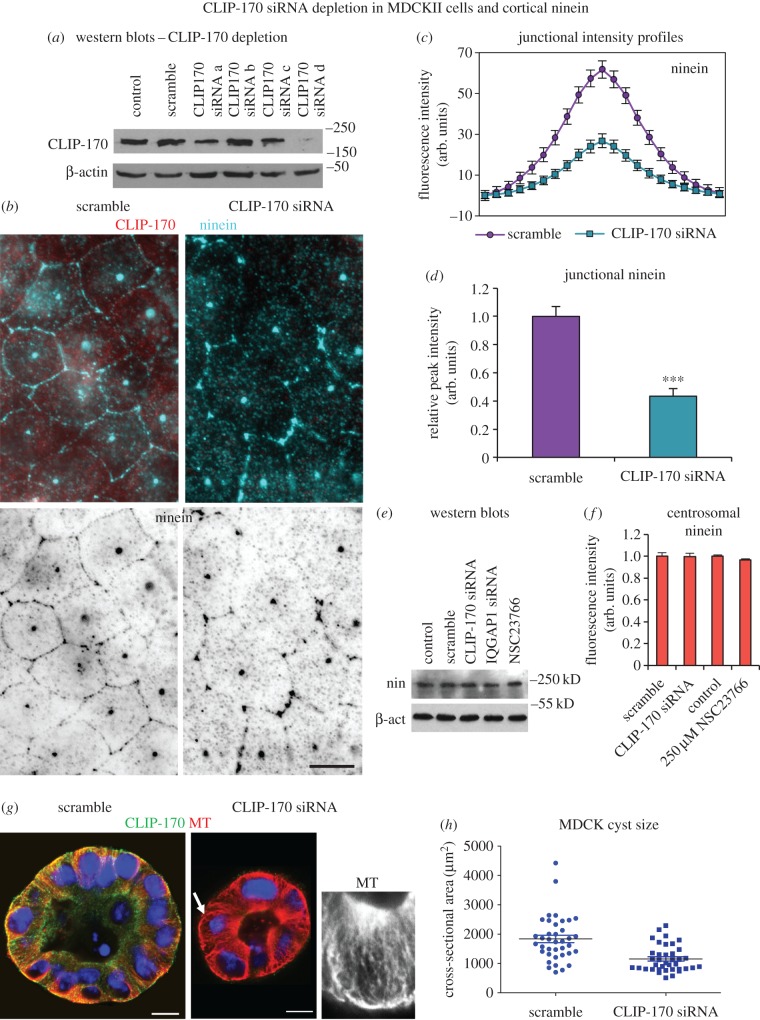
CLIP-170 siRNA knockdown in MDCKII cells leads to reduced cortical ninein and smaller cysts. (*a*) Western blot of lysates of control, scramble and canine CLIP-170 siRNA sequences (*a*–*d*) showing CLIP-170 and β-actin expression. (*b*) Scramble and CLIP-170 siRNA-treated cells fixed in methanol and stained for ninein (pAb N5, blue and invert) and CLIP-170 (mAb, red). (*c*) Junction fluorescence intensity profile analyses (*n* = 128) of ninein in scramble and CLIP-170-depleted cells. (*d*) Relative peak intensities of ninein at junctions in scramble and CLIP-170 siRNA-depleted cells reveal a significant decrease in ninein intensity in depleted cells (Mann–Whitney *U*-test, ***p < 0.05). (*e*) Western blot of lysates of control, scramble, CLIP-170 siRNA, IQGAP1 siRNA and Rac1 inhibitor NSC23766 (250 µM) treatments showing ninein (pAb Bethyl) and β-actin (pAb) expression. (*f*) Relative centrosomal ninein fluorescence intensity (*n* = 50) in control, scramble, CLIP-170 siRNA and Rac1 inhibitor NSC23766 (250 µM) treated cells revealing no significant difference (unpaired *t*-test). (*g*) Scramble and CLIP-170 siRNA-treated cells grown in Matrigel to induce cyst formation and fixed in formaldehyde–methanol and stained for MTs (mAb YL1/2, red) and CLIP-170 (pAb, green) at day 6 showing apico-basal MTs in both scramble and knockdown cysts. Note the marked decrease in cyst size in CLIP-170 siRNA-treated cysts. Inset shows MTs in depleted cell (arrow). (*h*) Cyst sizes in scramble and CLIP-170-depleted cells based on cross-sectional areas in μm^2^ with bars indicating averages showing significantly smaller cyst area in knockdown (Mann–Whitney *U*-test, ***p < 0.001). Scale bars, 10 µm.

In order to determine whether CLIP-170 affects cortical localization of ninein, it was knocked down using siRNA in MDCKII cells, which as previously described show distinct cortical n-MTOC ninein when confluent (partially polarized; figure 2*c*). Four CLIP-170 siRNA predicted sequences (*a*–*d*) were tested, with western blot analysis showing most efficient knockdown with sequence d (figure 4*a*). Confluent scramble siRNA control cells showed ninein at the centrosome, as speckles in the cytoplasm and at the cortex. However, CLIP-170 siRNA knockdown resulted in a marked reduction in cortical ninein (figure 4*b*). Average fluorescence intensity profiles through cell–cell junctions resulted in a 57% reduction in junctional ninein in CLIP-170 knockdown cells (figure 4*c*,*d*). Importantly, no differences in overall ninein protein levels or centrosome fluorescence intensity were observed between scramble and CLIP-170 siRNA-treated cells, showing that ninein expression and its dynamic exchange at the centrosome [8] had not been affected (figure 4*e*,*f*).

Both scramble and CLIP-170 siRNA knockdown MDCKII cells formed three-dimensional cysts with a central lumen and polarized cells (figure 4*g*). However, CLIP-170 knockdown resulted in markedly smaller cysts with 38.6% smaller cross-sectional area compared to scramble cysts (figure 4*h*).

### 2.5. *Clip1/Clip2* double knockout mouse intestine and organoids reveal abnormalities

**Figure 5.**
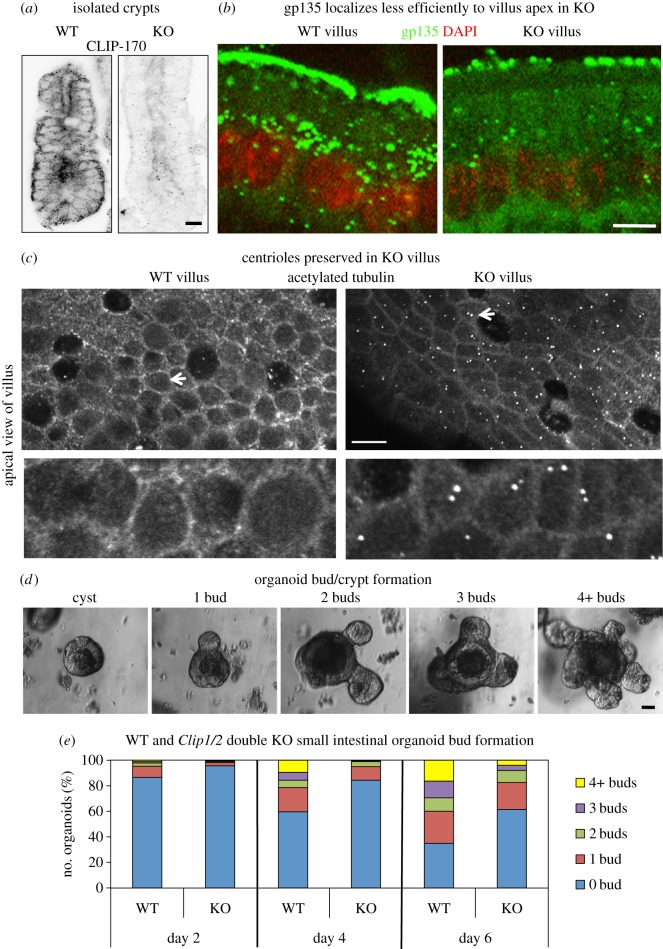
Small intestine of the *Clip1/Clip2* double knockout mouse. (*a*) Confocal optical sections of small intestinal crypts of WT and *Clip1/Clip2* KO mice fixed in formaldehyde–methanol and stained for CLIP-170 (pAb, invert) showing loss of CLIP-170 staining in knockout crypt. (*b*) Confocal images showing lateral views of paraformaldehyde fixed villus cells labelled for gp135 (rat mAb, green) and stained for DNA with DAPI (red) indicating markedly less apical gp135 in the KO compared with WT. (*c*) Optical sections at the level of the apical centrosome in WT and KO villus cells fixed in formaldehyde–methanol and labelled for acetylated tubulin (mAb) showing centrioles in KO cells but no evidence of centrioles in WT (arrows). The arrowed regions are enlarged in inset below. (*d*) Phase contrast images showing different stages of organoid (WT) development from cyst formation with no buds to fully formed organoids with several crypts (buds). (*e*) Graph showing the percentage of organoids with 0, 1, 2, 3 or 4 or more buds at day 2, 4 and 6 of development in organoids generated from WT and KO small intestine. Note that the formation of crypts (buds) is much slower in the KO compared to WT. Scale bars: (*a*,*c*) 10 µm, (*b*) 5 µm, (*d*) 20 µm. Two-way ANOVA statistical testing WT versus KO, day 2, day 4, day 6, *p* < 0.05.

The *in vitro* knockdown data suggested that CLIP-170 is required for efficient location of ninein to apical cortical n-MTOCs. The effect of lack of CLIP-170 was therefore investigated further in *ex vivo* intestinal tissue of the *Clip1/Clip2* double KO mouse in which the genes encoding CLIP-170 and CLIP-115, respectively, have been deleted (figure 5*a*). Although epithelia predominantly express CLIP-170, the double KO was used to prevent possible compensation by CLIP-115. *Clip1/Clip2* double KO mice survive, and the gross small intestinal morphology based on tissue sections appeared normal with the villus and crypts containing columnar epithelial cells (electronic supplementary material, figure S2). However, some developmental abnormalities were observed in both *ex vivo* tissue and *in vitro* organoids of the *Clip1/2* double KO.

**Figure 6.**
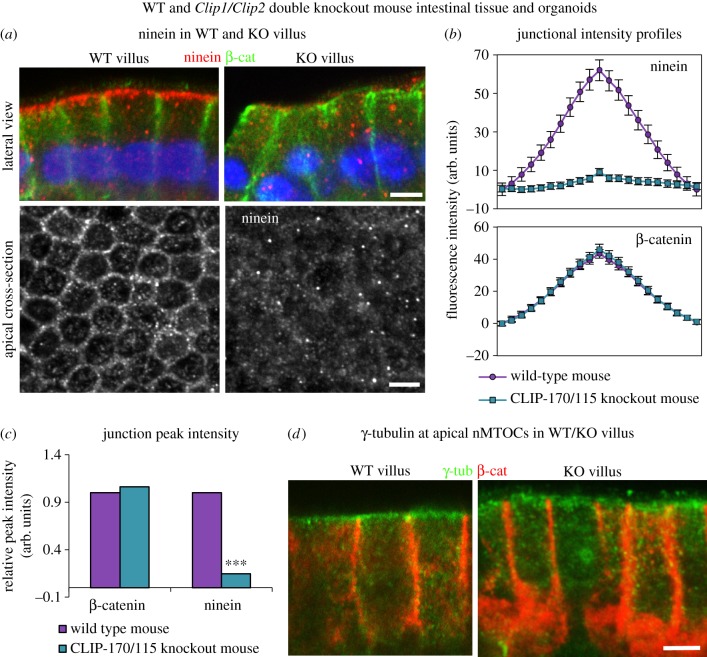
Loss of ninein at n-MTOCs in *Clip1/Clip2* double knockout mouse intestine. (*a*) Confocal images of methanol-fixed villus cells stained for ninein (pAb Pep3, red) and β-catenin (mAb, green) showing baso-lateral and apical cross-sectional views and revealing almost total absence of ninein at apical surface n-MTOCs in KO. (*b*) Fluorescence intensity profiles for β-catenin (*n* = 112) and ninein (*n* = 112) at junctions in WT and KO villus. (*c*) Relative peak fluorescence intensities for β-catenin and ninein at junctional sites in WT and KO villus revealing no significant difference in junctional β-catenin but a significant reduction in ninein (Mann–Whitney *U*-test, ***p < 0.001). (*d*) Confocal sections showing baso-lateral views of methanol-fixed villus cells stained for γ-tubulin (mAb, green) and β-catenin (pAb, red) revealing γ-tubulin at apical n-MTOCs in both WT and KO. Scale bars, 5 µm.

Epithelial cells with basal located nuclei and apico-basal MTs were evident in both crypt and villus tissue of the double KO (figures 5*b* and 6*a*; electronic supplementary material, figure S2). However, the apical polarity marker gp135 (podocalyxin) located more sparsely at the apical surface of villus cells in the KO compared to wild-type (WT) (figure 5*b*; electronic supplementary material, figure S3). Interestingly, in contrast to the WT, terminally differentiated KO villus cells retained their centrioles, suggesting that centriolar disassembly is affected in KO intestine (figure 5*c*). In addition, less acetylated MTs were apparent in the KO compared to WT villus cells (electronic supplementary material, figure S4).

Organoids were successfully generated from the small intestine of the *Clip1/2* double KO, but lack of CLIP-170/115 led to delayed development. Formation of buds that developed into crypts was significantly reduced in KO cultures compared to WT. There was a 43.5% increase in cysts with no buds and 75.9% fewer organoids with four or more buds in the KO compared to WT cultures by day 6 (figure 5*d*,*e*).

These findings suggest that although CLIP-170 is not essential for gut epithelial formation, it does appear to be required for efficient apical positioning of gp135, disassembly of the centrioles, maintenance of a population of acetylated MTs in terminally differentiated villus cells and efficient organoid development.

### 2.6. Intestinal epithelial cells from the *Clip1/Clip2* double knockout mouse lack ninein at apical n-MTOCs

Strikingly, almost complete absence of apical cortical ninein was evident throughout the small intestine of the *Clip1/2* double KO. Baso-lateral views of villus cells in the KO revealed a lack of apical cortical ninein, which was prominent in the WT (figure 6*a*, upper panels), with apical cross sections of the villus emphasizing the almost complete absence of ninein at the junctions (figure 6*a*, lower panel). Fluorescence intensity profiles through apical junctions showed an 86% reduction in ninein in the KO villus cells compared with the WT, while no change in β-catenin intensity confirmed that the junctions were intact (figure 6*b*,*c*).

γ-Tubulin is known to relocate to the apical surface n-MTOC in fully differentiated epithelial cells of the villus [11]. Interestingly, γ-tubulin showed similar apical surface location in KO compared to WT despite lack of ninein and CLIP-170 at the n-MTOCs, with fluorescence intensity analysis revealing no significant difference (figure 6*d* and data not shown). These results suggest that CLIP-170 is required for ninein but not γ-tubulin deployment to apical n-MTOCs during intestinal epithelial differentiation. In addition, it shows that γ-tubulin is not dependent on ninein for its localization at the apical surface n-MTOCs.

### 2.7. CLIP-170 siRNA depletion leads to reduced microtubule cortical targeting

**Figure 7.**
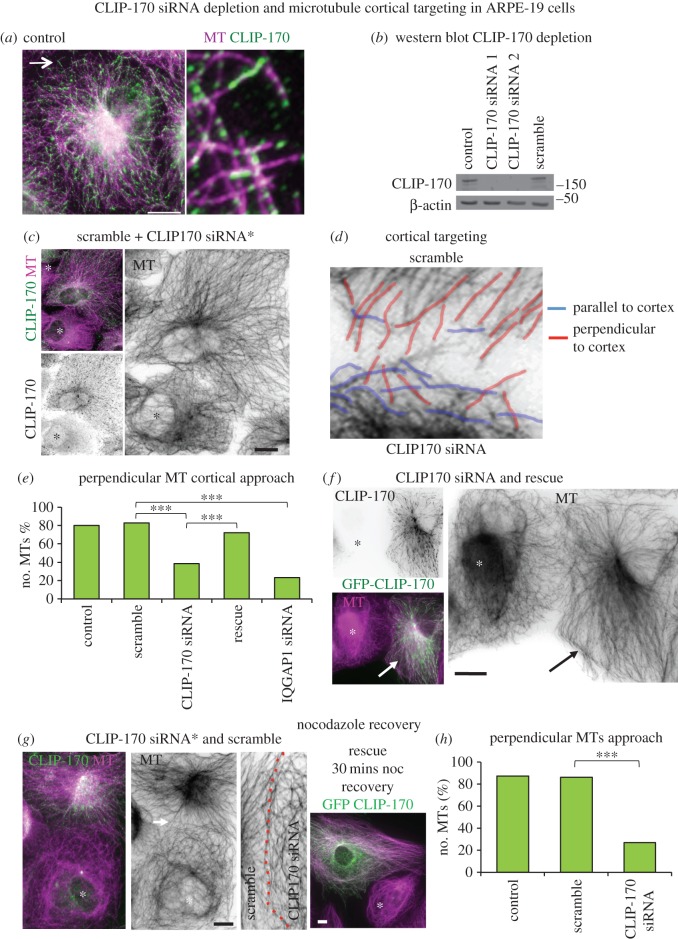
CLIP-170 siRNA depletion leads to compromised MT cortical targeting. (*a*) ARPE-19 cell methanol fixed and labelled for MTs (mAb YL1/2, purple) and CLIP-170 (pAb, green; enlarged region arrowed). (*b*) Western blot of lysates from control, scramble and CLIP-170 siRNA (human seq 1 and 2) ARPE-19 cells showing CLIP-170 (pAb) and β-actin expression. (*c*) Mixed culture showing a scramble cell next to a CLIP-170 siRNA-depleted cell (*) stained for CLIP-170 (pAb, green, invert) and MTs (mAb YL1/2, purple, invert). (*d*) Cell–cell contact between a scramble (top) cell and a CLIP-170-depleted (bottom) cell with perpendicular cortical targeting MTs highlighted in red and MTs parallel to the cell cortex in blue. (*e*) Graph showing the mean (*n* = 30) percentage of MTs with a perpendicular approach to cell–cell contacts in control, scramble, CLIP-170 siRNA, GFP-CLIP-170 rescue and IQGAP1 siRNA-treated cells. A non-parametric one-way ANOVA with Dunn's multiple comparison post-test was used and revealed no significance between control and scramble and between scramble and CLIP-170 rescue but significant differences between scramble and CLIP-170 siRNA, between scramble and IQGAP1 siRNA and between CLIP-170 siRNA and CLIP-170 rescue (***p < 0.001). (*f*) GFP-CLIP-170 (green, invert) expressing ARPE-19 cell (arrow) next to a CLIP-170-depleted cell (*), showing rescue of radial MT (purple, invert) organization. (*g*) Mixed culture of scramble and CLIP-170 siRNA (*) cells fixed 30 min following nocodazole removal and stained for MTs (purple, invert) and CLIP-170 green). The enlarged region of cell–cell contact (dotted red line) between scramble (right) and CLIP-170-depleted (left) cells shows lack of perpendicular MT approach in depleted cell. GFP-CLIP-170 (green) expressing ARPE-19 cell next to a CLIP-170-depleted cell (*) showing rescue of radial MT (purple) organization 30 min after nocodazole removal. (*h*) Graph showing the mean (*n* = 30) percentage of MTs with perpendicular approach to cell–cell contacts following nocodazole washout in control, scramble and CLIP-170 siRNA cells showing no significance between control and scramble but significant differences between control and CLIP-170 siRNA and between scramble and CLIP-170 siRNA (Mann–Whitney *U*-test, ***p < 0.001). Scale bars, 5 µm. Except for (*a*) 10 µm.

The CLIP-170 siRNA knockdown in MDCKII cells and *Clip1/2* KO mouse data revealed significant reductions in apical cortical ninein, suggesting that CLIP-170 is required for ninein deployment to n-MTOCs. This may be due to CLIP-170 facilitating MT plus-end cortical capture ensuring efficient delivery of ninein along MTs and/or due to cortical CLIP-170 recruiting ninein through cytoplasmic diffusion. We first determined whether MTs were involved in ninein redeployment to n-MTOCs. We established using a nocodazole assay and fluorescence intensity analysis that less cortical ninein was evident in confluent MDCKII cells following MT depolymerization while MT regrowth following nocodazole removal restored cortical ninein to control levels (data not shown). This suggests that MTs are required for efficient ninein localization to n-MTOCs. We then tested whether MT plus-end cortical targeting mediated by CLIP-170 is involved in ninein redeployment by siRNA depletion of CLIP-170 in human retinal pigmented epithelial cells (ARPE-19). ARPE-19 cells were chosen as they contain distinct radial arrays with MTs approaching the cortex perpendicularly, and any deviations from this pattern can easily be detected [6]. CLIP-170 localized as comets or elongated rods at plus-ends and as puncta along the MT lattice in ARPE-19 cells (figure 7*a*). Two different siRNA sequences against human CLIP-170 were used, both showing complete loss of CLIP-170 expression and no off-target effects on EB1 expression (figure 7*b*; electronic supplementary material, figure S5).

To better compare MT organization, mixed ARPE-19 cell cultures containing both depleted and scramble siRNA-treated cells were used. Cells were treated separately with either scramble or CLIP-170 siRNA and then mixed 24 h prior to immunolabelling. In the vast majority of CLIP-170-depleted cells, MTs had lost radial organization, centrosomal focus and perpendicular cortical approach (figure 7*c*). Many MTs appeared disorganized, forming a criss-cross pattern with several MTs aligned parallel to the cortex (figure 7*c*,*d*). To determine whether CLIP-170 has a role in MT cortical targeting, a perpendicular MT approach to the cortex was assessed blind in control, scramble and CLIP-170 siRNA-treated cells. Analyses showed a significant reduction in perpendicular MTs in CLIP-170-depleted cells compared to control/scramble cells (figure 7*e*). This could be rescued with GFP-rat-CLIP-170 that is not targeted by the siRNAs (figure 7*e*,*f*). MT cortical targeting was further assessed using a nocodazole regrowth assay in mixed cultures. MTs had fully recovered a radial array with a perpendicular cortical approach 30 min following nocodazole washout in control and scramble siRNA cells, whereas CLIP-170-depleted cells had not (figure 7*g*,*h*). Again this could be rescued with GFP-rat-CLIP-170 (figure 7*g*). This was also observed with CLIP-170 siRNA sequence 2 in U2OS cells (data not shown). These results suggest that MT cortical targeting is compromised in cells lacking CLIP-170 and this is likely to contribute to the reduced cortical ninein.

### 2.8. IQGAP1 acts as a cortical receptor for CLIP-170 and its knockdown leads to reduced cortical ninein

**Figure 8.**
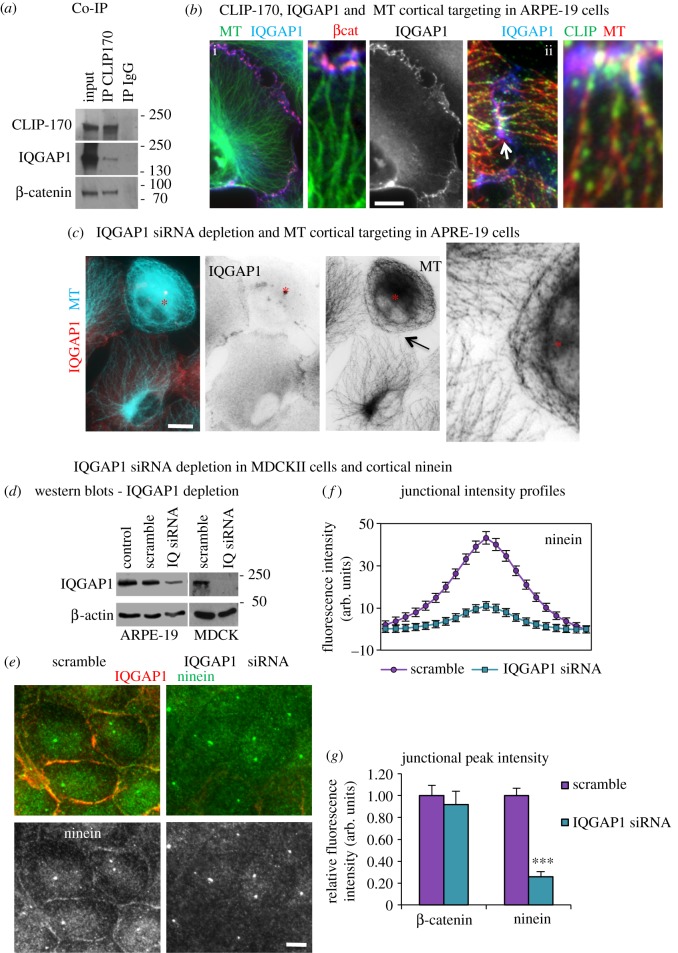
IQGAP1 siRNA depletion leads to loss of MT cortical targeting and reduced ninein at n-MTOCs. (*a*) Western blot of Co-IP experiments using either CLIP-170 or IgG as bait to pull down protein complexes in TC7 cells showing CLIP-170 pulls down endogenous CLIP-170, IQGAP1 and β-catenin but not the IgG control lanes (CLIP-170 pAb was used for probing but mAb used as bait). (*b*) ARPE-19 cells methanol fixed and stained for IQGAP1 (mAb), MTs (YL1/2) and β-catenin (pAb), purple in *b*(i) indicating colocalization and nocodazole recovery. (*b*(ii)) showing CLIP-170-bound MTs targeting cortical IQGAP1 located on the inner face of junctional β-catenin puncta. The arrow indicates region enlarged in the inset to the left. (*c*) Mixed culture of ARPE-19 cells fixed in methanol showing a scramble cell next to a IQGAP1-depleted cell (*) stained for IQGAP1 (mAb, red, invert) and MTs (rab α-tubulin, blue, invert). Enlarged region (arrow) showing lack of cortical MT targeting in IQGAP1-depleted cell. (*d*) Western blots of lysates of control, scramble and IQGAP1 siRNA ARPE-19 and MDCKII cells showing IQGAP1 and β-actin expression. (*e*) Scramble and IQGAP1 siRNA-treated cells methanol fixed and stained for ninein (pAb Pep3, green) and IQGAP1 (mAb, red) showing less cortical ninein in depleted cells. (*f*) Junctional fluorescence intensity profile (*n* = 92) for ninein in scramble and IQGAP1 siRNA-treated cells. (*g*) Relative peak fluorescence intensities for β-catenin and ninein at junctions in scramble and IQGAP1 siRNA-treated cells showing no significance in β-catenin intensities (unpaired *t*-test) but a significant reduction in ninein (non-parametric Mann–Whitney *U*-test, ***p < 0.001). Scale bars, 10 µm except (*b*(ii)), 2 µm.

The cortical receptor and Rac1/Cdc42 effector IQGAP1 has been shown to interact with CLIP-170 and to capture and stabilize MTs at the cell cortex in migrating cells [31]. However, its role in MT capture at cell junctions has not been investigated. Here, we show that IQGAP1 coimmunoprecipitated with CLIP-170 in confluent human intestinal TC7 cells, suggesting that these proteins also interact in non-migrating epithelial cells (figure 8*a*). In addition, the CLIP-170 IP also pulled down the AJ component β-catenin (figure 8*a*), which has also been reported to interact with IQGAP1 [38].

IQGAP1 localized to the inner face of β-catenin puncta at cell–cell contacts in ARPE-19, MDCKII, HeLa, TC7 and U2OS epithelial cells with MTs directly targeting IQGAP1/β-catenin clusters (figure 8*b* and data not shown). Nocodazole recovery assays in ARPE-19 cells showed re-forming MTs positive for CLIP-170 target cortical IQGAP1 at cell–cell junctions (figure 8*b*(ii)). IQGAP1 may thus act as a cortical receptor at AJs for the capture of CLIP-170-bound MTs and facilitate ninein relocation. Depletion of IQGAP1 in ARPE-19 cells produced similar results to CLIP-170, with a significant reduction in MT perpendicular approach, suggesting that IQGAP1 influences MT plus-end targeting/capture (figures 7*e* and 8*c*).

Previously, IQGAP1 expression has been linked to junction integrity, which in turn could affect cortical ninein accumulation [39]. The maintenance of junction integrity was confirmed by IQGAP1 knockdown in MDCKII cells, with fluorescence intensity profiles through cell junctions revealing no change in β-catenin (figure 8*g*). However, a marked loss in cortical ninein was evident in IQGAP1 siRNA-treated cells with junctional fluorescence intensity profiles showing a 74% reduction in ninein (figure 8*e*–*g*) despite the total ninein protein level remaining the same (figure 4*e*). This suggests that IQGAP1 coordinates with CLIP-170 to mediate MT cortical targeting and capture to facilitate ninein redeployment.

### 2.9. Rac1 inhibition affects MT dynamics and cortical targeting and leads to reduced cortical ninein

Active Rac1 has been reported to promote CLIP-170 and IQGAP1 complex formation and to facilitate and prolong MT cortical capture in migrating cells, with active Rac1 promoting MT growth into lamellipodia [31,40]. However, the effect of Rac1 on MT organization and dynamics in confluent epithelial cells is not known and was therefore investigated with regard to ninein redeployment.

**Figure 9.**
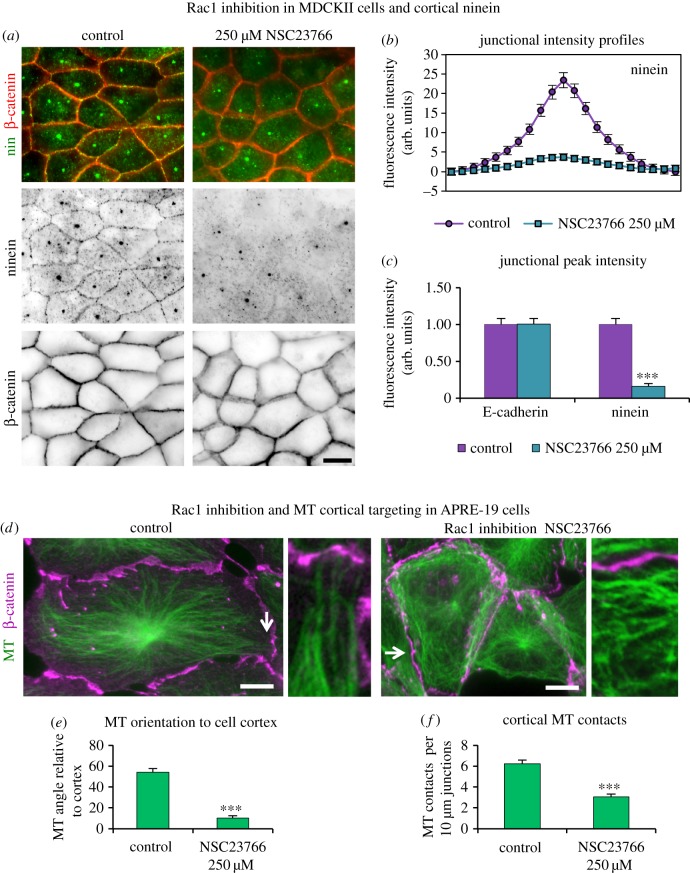
Rac1 inhibition leads to reduced cortical ninein and MT junctional targeting. (*a*) Control and Rac1-inhibited (250 µM NSC23766) cells methanol fixed and stained for ninein (pAb N5, green, invert) and β-catenin (mAb, red, invert) showing a marked reduction in cortical ninein in Rac1-inhibited cells. (*b*) Junctional fluorescence intensity profile for ninein (*n* = 112) in control and Rac1-inhibited cells. (*c*) Relative peak fluorescence intensity of E-cadherin and ninein at junctions in control and Rac1-inhibited cells showing no significance in E-cadherin but in ninein (non-parametric Mann–Whitney *U*-test, ***p < 0.001). (*d*) Control and Rac1-inhibited (250 µM NSC23766) ARPE-19 cells methanol fixed and stained for β-catenin (mAb, purple) and MTs (pAb α-tubulin, green), with enlarged regions (arrowed) highlighting cortical MT approaches. Note several MTs aligned parallel to the cortex in Rac1-inhibited cells. (*e*) Graph showing the mean MT orientation to cell junctions (*n* = 30), using FibrilTool [42] revealing significant (***) deviation from perpendicular targeting in inhibited cells (non-parametric Mann–Whitney *U*-test, ***p < 0.001). (*f*) Graph showing the mean (*n* = 30) number of MT contacts per 10 µm, junctional β-catenin staining revealing significantly fewer cortical contacts in inhibited cells (unpaired *t*-test, ***p < 0.001). Scale bars, 10 µm.

Rac1 was evident at AJs in MDCKII and APRE-19 cells colocalizing with β-catenin, IQGAP1 and ninein (electronic supplementary material, figure S6*a*). Interestingly, Rac1 aligned along MTs in some junctional regions (electronic supplementary material, figure S6*b*). Rac1 was inhibited with NSC23766, a specific inhibitor of Rac1–GEF interaction that prevents Rac1 activation [41]. Loss of peripheral actin arcs and dorsal stress fibres (perpendicular actin bundles) and an increase in ventral stress fibres in NSC23766-treated cells confirmed effective Rac1 inhibition (electronic supplementary material, figure S6*c*). The effect of Rac1 inhibition on ninein redeployment was investigated in MDCKII cells. Cells treated with NSC23766 showed 84% reduction in cortical ninein compared to control cells (figure 9*a*–*c*). Fluorescence intensity profiles of E-cadherin confirmed the maintenance of junction integrity and showed unchanged centrosomal ninein and protein levels in Rac1-inhibited cells (figures 4*e*,*f* and 9*c*). Reduced cortical ninein in Rac1-inhibited cells suggests that active Rac1 promotes efficient ninein delivery and that MT cortical targeting and dynamics may be affected by Rac1 inhibition.

Rac1 inhibition in confluent ARPE-19 cells maintained centrosome focused MT arrays but resulted in extensive looping around the cell periphery (figure 9*d*). To quantify MT cortical targeting and approach, the relative orientation of MTs to cell junctions was assessed using the ImageJ (FIJI) plugin ‘FibrilTool’ [42]. In control cells, the MTs were on average orientated at 54° to the cell junctions, which was reduced to 10° in Rac1-inhibited cells, confirming a close-to-parallel orientation (figure 9*e*). Analysis of MT cortical contact, assessed by counting the number of contacts per 10 µm of junction, revealed on average six MT contacts in control cells but only three in Rac1-inhibited cells (figure 9*f*). These data suggest that active Rac1 is required for cortical MT targeting and contact at cell junctions.

**Figure 10.**
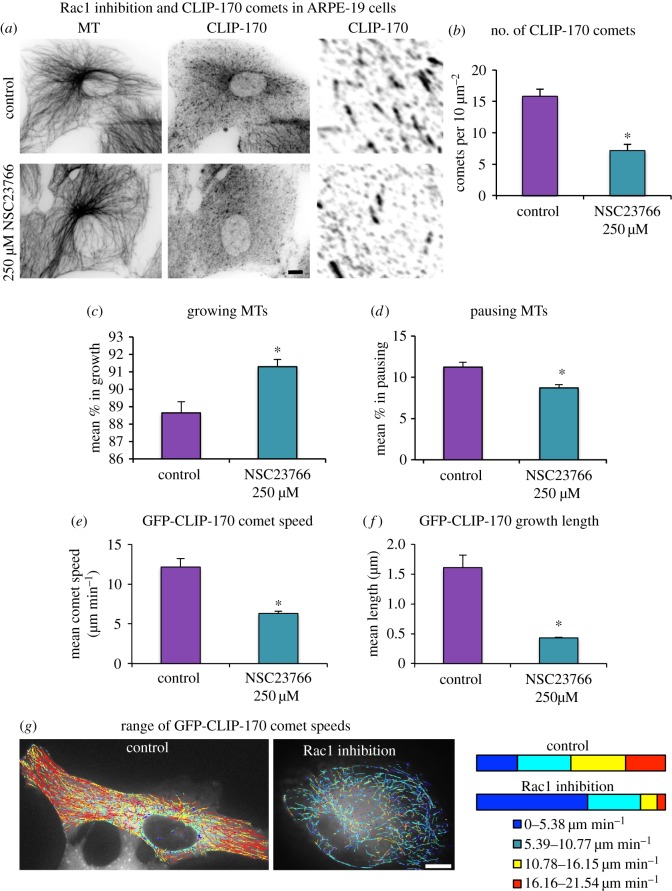
Rac1 inhibition leads to fewer and slower CLIP-170 comets and decreased pausing events. (*a*) Control and Rac1-inhibited (250 µM NSC23766) ARPE-19 cells fixed in formaldehyde–methanol and stained for CLIP-170 (pAb) and MTs (mAb YL1/2) with enlargements of comets. (*b*) Graph showing the mean number of CLIP-170 comets (*n* = 30) for each treatment showing a reduction in comets with Rac1 inhibition. (*c*–*g*) GFP-CLIP-170 dynamics in control and Rac1-inhibited ARPE-19 cells. (*c*,*d*) The mean (*n* = 4) percentage of composite tracks defined as growing or pausing. Only top part of graph is shown in (*c*). (*e*,*f*) Analysis of the mean (*n* = 4) GFP-CLIP-170 comet speed and growth length. (*g*) Plots of GFP-CLIP-170 growth tracks colour coded according to speed with bar plot showing the mean (*n* = 4) percentage of tracks in each speed group. See also the electronic supplementary material, movies S1 and S2. Scale bars, 10 µm. (*b*–*f*) Non-parametric Mann–Whitney *U*-test, *p < 0.05.

To assess the effect of Rac1 inhibition on MT dynamics, the number of CLIP-170 comets was analysed. Rac1 inhibition led to significantly fewer CLIP-170 comets (figure 10*a*,*b*). MT dynamic behaviour was further studied in ARPE-19 cells expressing GFP-CLIP-170 by live time-lapse image analysis using the automated tracking software U-Track, originally plusTipTracker [43]. It should be noted that stabilized MTs will generally not be detected by this method and the addition of GFP-CLIP-170 may promote some MT rescue. GFP-CLIP-170 comet analysis showed more growth and fewer pausing events in Rac1-inhibited cells compared to control cells (figure 10*c*,*d*; electronic supplementary material, movies S1 and S2). However, the phases of growth were shorter and the average comet velocity was lower in Rac1-inhibited cells (6.3 µm min^−1^) compared to control (12.1 µm min^−1^) cells (figure 10*e*,*f*). To further analyse the differences in growth, the data were divided into four speed groups and the distribution for each treatment was studied. The Rac1-inhibited cells showed a different distribution of comet speeds with reduced fast and very fast comets but increased percentages of very slow comets (figure 10*g*). This suggests that active Rac1 encourages fast persistent MT growth to the AJs and initiates MT capture by promoting pausing.

### 2.10. Apico-basal microtubule arrays are maintained in the absence of ninein at n-MTOCs

**Figure 11.**
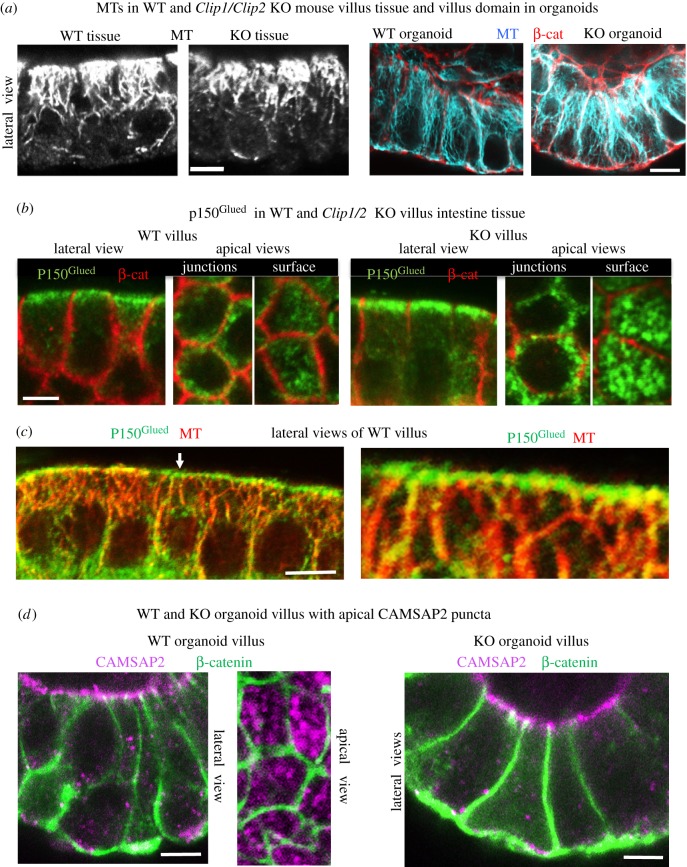
Apico-basal MTs and CAMSAP2 and p150^Glued^ at n-MTOCs in both WT and KO villus cells. (*a*) Formaldehyde–methanol-fixed isolated villus epithelial tissue (right) stained for MTs (mAb YL1/2) and organoid villus-domain epithelial cells (left) stained for MTs (blue) and β-catenin (pAb, red) showing apico-basal MTs in both WT and KO. (*b*) Villus stained for p150^Glued^ (mAb, green) and β-catenin (pAb, red) showing apical surface and junction localization in both WT and KO. (*c*) Isolated WT villus tissue labelled for p150^Glued^ (mAb, green) and MTs (pAb α-tubulin, red) showing apical concentration of 150^Glued^ at n-MTOCs and apico-basal MTs with minus-ends targeting p150^Glued^ puncta (arrow indicates enlarged area to right). (*d*) Organoid villus-domain cells stained for CAMSAP2 (pAb, purple) and β-catenin (mAb, green) showing CAMSAP2 puncta at apical surface n-MTOCs in organoids generated from both WT and KO small intestine. Scale bars, 5 µm.

Here, we have shown that ninein expression is essential for apico-basal MT formation and epithelial elongation, and that ninein recruitment to apical n-MTOCs is dependent on CLIP-170 and its coordination with IQGAP1 and active Rac1. However, apico-basal MT organization was evident in villus cells of both *ex vivo* intestinal tissue and *in vitro* organoids of the KO as well as in CLIP-170 knockdown MDCKII cysts (figures 4*g* and 11*a*). Note that ninein is present at the centrosome and as speckles in the cytoplasm in both KO and knockdown cells, and the level of its expression is unchanged (figure 4*e*). The presence of apico-basal MTs despite lack of ninein at the apical n-MTOCs in knockdown/KO epithelial cells reveals that ninein is not essential for apico-basal array maintenance and suggests that it is not essential for MT minus-end anchorage at n-MTOCs and that other proteins/complexes compensate. Indeed, the dynactin subunit p150^Glued^, which has been reported to have a role in MT minus-end anchorage at the centrosome [44], was evident at the apical n-MTOCs in both WT and KO villus cells (figure 11*b*,*c*). Cross sections of villus cells revealed p150^Glued^ puncta at apical junctions and surfaces in both KO and WT while lateral views indicated MT minus-ends within apical p150^Glued^ puncta (figure 11*b*,*c*). Most interesting, CAMSAP2, a member of the novel calmodulin-regulated spectrin-associated protein family, which binds and stabilize the minus-ends of non-centrosomal MTs [45–48], was also evident at apical n-MTOCs in WT villus tissue colocalizing with p150^Glued^ (electronic supplementary material, figure S7). Furthermore, CAMSAP2 was present at apical n-MTOCs in *in vitro* organoids generated from both WT and KO small intestine (figure 11*d*).

The results reveal that although ninein expression is required for apico-basal MT formation and epithelial polarization, its localization at n-MTOCs is not essential for maintenance of the apico-basal array. Furthermore, the findings show that CLIP-170 is required for ninein but not γ-tubulin, p150^Glued^ or CAMSAP2 deployment to apical n-MTOCs during intestinal epithelial differentiation.

## 3. Discussion

How the centrosome reorganizes its components and how n-MTOCs form are poorly understood, and yet n-MTOCs are critical for apico-basal epithelial differentiation as they determine MT positioning, which underpins cell shape and function [4,49,50]. Here, we show using both *in vitro* siRNA depletion and *ex vivo* mouse KO studies that CLIP-170 is required for redeployment of the MT minus-end anchoring protein ninein to n-MTOCs but not for γ-tubulin. The data suggest that CLIP-170 together with IQGAP1 and Rac1 form a complex at AJs that facilitates ninein relocation to n-MTOCs during differentiation. Loss of CLIP-170 delayed development of three-dimensional epithelial and mouse organoid cultures, although lack of ninein at n-MTOCs did not prevent the formation and maintenance of apico-basal arrays. Interestingly, p150^Glued^ and the novel MT minus-end stabilizer CAMSAP2 maintained their location at n-MTOCs in *Clip1/Clip2* double KO organoids and may compensate for the lack of ninein. In addition, although ninein is able to bind γ-tubulin [20], the KO data revealed that γ-tubulin is not dependent on ninein for its localization at apical surface n-MTOCs. This is also the case for *Caenorhabditis elegans*, where γ-tubulin is recruited to n-MTOCs independently of the ninein-related protein NOCA-1 [51].

A defining step in epithelial differentiation is the accumulation of anchoring proteins such as ninein at apical n-MTOCs while centrosomal MT anchorage diminishes [8]. The present study showed that fully differentiated epithelial cells in cysts and small intestinal *ex vivo* tissue and *in vitro* organoids possess ninein and CLIP-170 at n-MTOCs, which are associated with AJs in MDCKII and with both AJs and the apical surface in intestinal villus cells. In contrast, in proliferating cells located in the stem cell region at the bottom of the intestinal crypts, ninein was concentrated at the apical centrosome while CLIP-170 was present as MT plus-end comets in the cytoplasm. We have previously established that ninein is highly dynamic and moves in and out of the centrosome in a MT-dependent manner [8], and here we further show that MTs are required for ninein redeployment to n-MTOCs. In addition, nocodazole assays and ultrastructural analyses have suggested that initial MT plus-end targeting followed by minus-end anchorage at AJs are important steps in the generation of non-centrosomal apico-basal MTs [6]. In the present study, depletion or inhibition of CLIP-170, IQGAP1 or Rac1 caused compromised MT cortical targeting and a dramatic reduction in ninein location at n-MTOCs.

Both CLIP-170 and EB1 have been implicated in MT cortical targeting for adherens and gap junction formation [29,30]. Here, we have shown that CLIP-170-bound MT plus-ends target IQGAP1 puncta on the inner face of β-catenin at cell–cell contacts with their interaction confirmed by Co-IP. The localization of IQGAP1 at AJs is likely to be mediated through its binding to β-catenin, E/N-cadherin, active Rac1 or F-actin [38,52,53]. IQGAP1 is thus ideally positioned at AJs to capture MT plus-ends via CLIP-170, although β-catenin has also been identified as an interactor of CLIP-170 and may act as an alternative cortical receptor. Depletion of either CLIP-170 or IQGAP1 resulted in loss of MT cortical targeting as well as a significant reduction in cortical ninein. The importance of CLIP-170 in ninein redeployment during epithelial differentiation was also verified in *ex vivo* intestinal tissue and organoids of the *Clip1/Clip2* double KO mouse, which lack CLIP-170 and CLIP-115 and fail to locate ninein to n-MTOCs.

Rac1 inhibition resulted in fewer CLIP-170 comets, increased MT stability, compromised cortical targeting and a significant reduction in cortical ninein. This fits with other studies where Rac1 inhibition has been shown to suppress MT dynamics and targeted growth as, for example, in fibroblasts [54]. In endothelial cells, active Rac1 is required for IQGAP1, EB1 and cortactin complex formation and MT cortical capture [55]. A more detailed analysis of MT dynamics using live GFP-CLIP-170 imaging and U-Track revealed more growth and fewer pausing events, with a significant reduction in fast comets in inhibited cells compared with control cells. This suggests that active Rac1 facilitates ninein relocation by promoting fast persistent MT growth towards AJs, with increased pausing enabling capture at these sites. Interestingly, RNAi knockdown of EB1 in epithelial cells, using a previously characterized EB1 shRNA [24], had no effect on cortical ninein relocation (data not shown), suggesting that specific +TIP capturing complexes are required for ninein redeployment.

**Figure 12.**
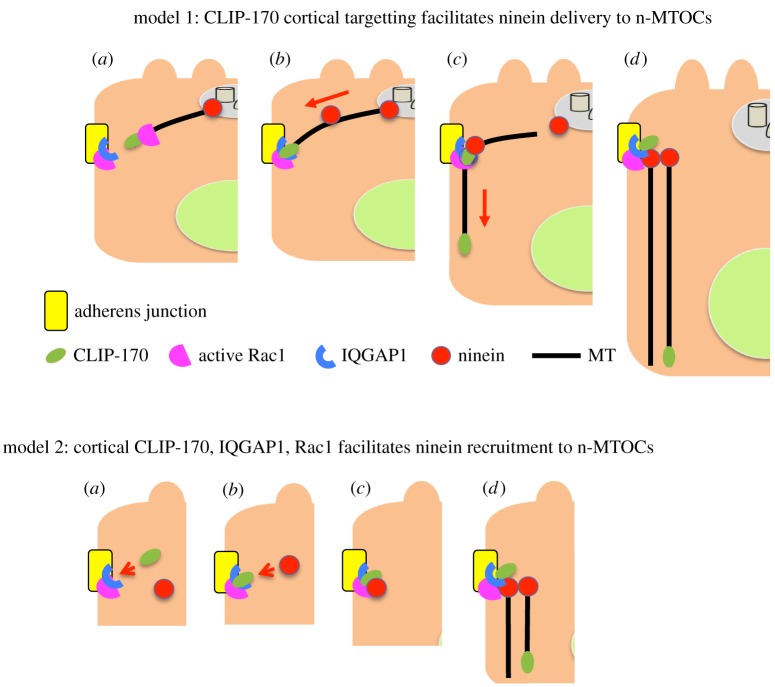
Models for ninein redeployment to n-MTOCs during epithelial differentiation. Model 1: (*a*) CLIP-170 (green) bound MTs elongate and target IQGAP1 (blue) at adherens junctions (yellow) in a process promoted by active Rac1 (pink). (*b*) CLIP-170, IQGAP1 and active Rac1 facilitate MT capture at adherens junction-associated n-MTOCs, and ninein (red) is transported along MTs. (*c*) Ninein and CLIP-170 bind to adherens junctions, MT minus-ends are released from centrosome and plus-ends elongate towards the cell base. (*d*) Ninein anchors MT minus-ends at n-MTOCs at adherens junctions while plus-ends elongate towards cell base thus generating the apico-basal array. Model 2: (*a*) CLIP-170 (green) is recruited to apical adherens junctions (yellow) and forms a complex with IQGAP1 (blue) and active Rac1 (pink). (*b*) Cortical receptor complex IQGAP1, CLIP-170 and active Rac1 recruits ninein (red) to apical adherens junctions. (*c*) Ninein accumulates at forming n-MTOCs associated with apical adherens junctions. (*d*) MT (black) minus-ends are captured by ninein at n-MTOCs and plus-ends elongated towards the cell base.

We propose two alternative but not mutually exclusive models for ninein redeployment to n-MTOCs. In the first model, CLIP-170-bound MT plus-ends target and are captured by IQGAP1 at apical AJ associated n-MTOCs in a process promoted by active Rac1. Here, CLIP-170 acts as a +TIP facilitating MT cortical targeting and ninein delivery along MTs to the n-MTOCs. Loss of CLIP-170, IQGAP1 or active Rac1 results in compromised MT cortical targeting/capture and ninein delivery to n-MTOCs. In the second model, CLIP-170 together with IQGAP1 and active Rac1 act as a cortical receptor/anchoring complex for ninein at n-MTOCs with loss of CLIP-170, IQGAP1 or active Rac1 resulting in defective ninein recruitment to the n-MTOCs. Future dynamic analyses of MTs and ninein will be needed to determine the exact mechanism by which ninein is localized to n-MTOCs (figure 12).

It is well established that ninein is essential for MT minus-end anchorage at the centrosome, but its role in formation and maintenance of non-centrosomal apico-basal MT arrays had not been investigated [19,56]. Here, we show for the first time that expression of ninein and most likely its presence at the centrosome and/or in the cytoplasm as speckles are required for apico-basal array formation and columnar epithelial differentiation. Similarly, the ninein-related protein NOCA-1 in *C. elegans* has also been found to be required for assembly of non-centrosomal MT arrays in epithelial cells [51]. Lack of ninein maintained the undifferentiated epithelial phenotype of relatively flat cells with disorganized MT networks. The centrosomal protein CAP350 has also been reported to influence apico-basal MT organization and epithelial elongation. However, CAP350 does not localize to the apical n-MTOCs but to baso-lateral junctions and assists MT bundle formation by facilitating MT adherens junction interactions [57,58].

Interestingly, knockdown of CLIP-170 or loss of *Clip1/2* gene expression did cause noticeable developmental abnormalities including reduced cyst size and delayed gut organoid development. In particular, less efficient apical distribution of the transmembrane glycoprotein and polarity marker gp135 (podocalyxin) and fewer acetylated MTs suggest that apical transport and MT stability are affected in the KO and this is likely to be linked to lack of CLIP-170. MTs play an important role in the delivery of gp135, while binding of CLIP-170 along the MT lattice as observed in WT villus cells has been linked to increased MT stability and tubulin acetylation in other cell types [59–63]. Interestingly, centriole disassembly is also affected, and future analysis will be needed to determine whether CLIP-170 and/or ninein redeployment play a role.

Surprisingly, anchorage of apico-basal MTs at n-MTOCs in differentiated epithelial cells is not dependent on ninein or CLIP-170 as knockdown of CLIP-170 in cells and KO of the *Clip1/2* genes in mouse intestine prevented ninein localization to n-MTOCs but not epithelial elongation or apico-basal MT formation and maintenance. Loss of desmoplakin in the villus has also been reported to affect apical ninein localization without affecting the formation of columnar epithelial cells or apico-basal MT arrays [64]. This suggests that other anchoring proteins compensate for lack of ninein at n-MTOCs. Indeed, p150^Glued^, which has been implicated in centrosomal anchoring [44], remained at the n-MTOCs in KO villus cells. Most interesting, CAMSAP2, a member of the novel calmodulin-regulated spectrin-associated protein family, which binds and stabilizes the minus-ends of non-centrosomal MTs, was evident at apical n-MTOCs in *ex vivo* intestinal villus tissue [45,48,65,66]. Furthermore, CAMSAP2 also localized to n-MTOCs in organoids generated from both WT and *Clip1/2* double KO small intestine and thus in the presence or the absence of ninein and CLIP-170 at the n-MTOCs. CAMSAP3 has recently been identified as important for tethering MTs to the apical cortex in intestinal cells, with depletion or mutations disrupting MT organization although without loss of overall apico-basal orientation [67]. However, loss of either CAMSAP2 or CAMSAP3 has no effect on formation of polarized intestinal epithelial cysts in three-dimensional culture although loss of non-centrosomal MTs are apparent in CAMSAP3 but not CAMSAP2 KO cells in two-dimensional polarizing cultures [68]. Taken together, this suggests that the minus-ends of apico-basal MTs are anchored to n-MTOCs by multiple complexes, with loss of ninein from the n-MTOCs compensated for by others such as CAMSAPs. This is particularly interesting as in *C. elegans* the ninein homologue NOCA-1 functions redundantly with PTRN-1 (CAMSAP homologue) in the assembly of non-centrosomal MT arrays in some tissues [51]. It therefore seems likely that both MT minus-end anchoring proteins such as ninein and stabilizing proteins such as the CAMSAPs cooperate and are recruited to n-MTOCs to maintain non-centrosomal MT arrays. Proteins that act as platforms for the recruitment of MT minus-end nucleating and/or anchoring/stabilizing proteins are also likely to be important for assembly of n-MTOCs. Here, our data suggest that IQGAP1/Rac1 and CLIP-170 act as a platform at apical AJs for the recruitment of ninein and formation of anchoring n-MTOCs in differentiating kidney epithelial cells (MDCKII). Interestingly, the spectraplakin ACF7 (MACF1) has recently emerged as critical for the recruitment of CAMSAP3-bound MTs to apical surface n-MTOCs and for the formation of polarized intestinal epithelial cysts [68]. In *Drosophila*, the homologue of ACF7, Shortstop (Shot) and Patronin (CAMSAP homologue) localize to apical domains together with spectrin and cooperate to generate MT array [69]. Further studies will be required to determine the exact role and interplay of these components in non-centrosomal MT minus-end anchorage at n-MTOCs.

## 4. Material and methods

### 4.1. *Clip1/Clip2* double knockout mouse

Generation of the *Clip1/Clip2* double KO mouse strain will be described elsewhere. Briefly, the genes encoding CLIP-170 (*Clip1*) and CLIP-115 (*Clip2*) were targeted as described [59,70]. The *Clip1* gene was subsequently further modified in embryonic stem cells to obtain a completely deleted gene. *Clip1* and *Clip2* single KO mice were then crossed to generate the double KO line. Mice were maintained on a C57Bl6 background by crossing heterozygous double KO mice with WT C57Bl6 animals (obtained from Harlan, NL). To obtain homozygous double KO mice for actual experiments, heterozygous male and female mice were mated, and the F1 offspring used. The WT mice used in these studies were all littermates of the homozygous KO animals and at P40–80.

### 4.2. Cell culture and drug treatment

ARPE-19 (human retinal pigment epithelial) cells were cultured in DMEM/F12 (Invitrogen) medium supplemented with 10% FBS (Invitrogen), 1% l-glutamine (Invitrogen) and 2% sodium bicarbonate (Invitrogen) at 37°C in 5% CO_2_. U2OS (human osteosarcoma), TC7 (human colorectal carcinoma) and MDCKII (Madin–Darby canine kidney) cells were cultured in DMEM (Invitrogen) containing 10% FBS, 1% l-glutamine and 0.1 mg ml^−1^ streptomycin and 100 units ml^−1^ penicillin. MDCKII cells were seeded in Matrigel (Corning) and grown for 6 days for three-dimensional cyst experiments.

Nocodazole assays were performed as previously described [6]. Inhibition of Rac1 activation was performed using the chemical inhibitor NSC23766 (Tocris; effectiveness between 10 and 1000 µM [41]). For Rac1 inhibition, confluent ARPE-19 and MDCKII cells were treated with 250 µM NSC23766 for 12 or 24 h, respectively.

Organoids from WT and KO mice were established as previously described [37], and both were maintained for 3+ months in culture. For budding experiments, organoids were digested with TrypLE express (Invitrogen) for 3 min at 37°C and fragmented by pipetting. These fragments were then seeded in Matrigel and maintained under normal organoid culturing conditions [37].

### 4.3. Immunolabelling and antibodies

Fixation and immunolabelling of cultured cells were performed as previously described [6]. Small intestine was isolated and fractioned as previously described [22,71,72]. Isolated fractions were fixed in cold (−20°C) methanol or formaldehyde (9%)/methanol for 10 min and stained as above. Organoid and cyst were fixed either in their Matrigel setting or following extraction by Cell Recovery Solution (Corning) and then subsequently immunostained as previously described [22].

Rabbit polyclonal antibodies against β-catenin (Sigma) were used at 1 : 2000, ninein Pep3 [8] at 1 : 1000, CAMSAP2 (CAMSAP1L1, Proteintech) at 1 : 500 and α-tubulin (Abcam ab15246), IQGAP1 H-109 (Santa Cruz), ninein N5 (Abcam ab52473) and CLIP-170 2360 [73] at 1 : 200. Mouse monoclonal antibodies against β-catenin (BD Biosciences) and γ-tubulin (Abcam ab11316) were used at 1 : 1000, E-cadherin (BD Biosciences) at 1 : 500, IQGAP1 (BD Biosciences), Rac1 (BD Biosciences), p150^Glued^ (BD Biosciences), dynein intermediate chain 70.1 (Sigma) and acetylated tubulin (Sigma) at 1 : 200 and CLIP-170 F3 (Santa Cruz) at 1 : 50. Rat monoclonal antibodies against tyrosinated tubulin clone YL1/2 (Abcam ab6160) and GP135/Podocalyxin (R&D Systems mab1556) were used at 1 : 1000 and 1 : 50, respectively. Secondary antibodies conjugated to Alexa-Fluor 488, 568 or 647 (Invitrogen) were used at 1 : 1000 and DAPI (Sigma) at 1 : 2000. Highly cross-absorbed secondary antibodies conjugated to Dylight-488 and 647 (Jackson) were used at 1 : 800. Phalloidin conjugated to Alexa-488 (Invitrogen) was used at 1 : 200 for labelling of actin filaments.

### 4.4. siRNA and cDNA transfection

ARPE-19 and U2OS cells were treated with 27 nM of siRNA (Qiagen) delivered by Oligofectamine (Invitrogen) as per the manufacturer’ protocol at 0 h and again at 48 h, with experiments performed at 96 h. Mixed cultures were generated by passaging cells at 72 h and mixing siRNA-treated with scramble, or untreated control cells, and then seeding them onto coverslips. For siRNA knockdown in MDCKII cells (1 × 10^6^), 200 pmol of siRNA was delivered using Amaxa (Lonza) electroporation programme A-23 at 0 h and again at 48 h. At 60 h, cells were seeded confluent (0.3 × 10^6^ cells per 10 mm coverslip) then lysed or immunostained at 96 h. TC7 cell depletion in polarized cells was performed and analysed as previously described [22].

Allstars scramble siRNA (Allstar, Qiagen) was used for all siRNA negative controls. Human ninein target sequences: seq a GCCAGGGTTAGTAATGTCTTCTTGT [15], seq. 2 CGGTACAATGAGTGTAGAA [8] and seq. 3 GGAAGACCTAAGAAATGTA [8]. Human CLIP-170 target sequences: seq. 1 CCCGACCTTCAAAGTTAACAA, seq. 2 CCCGTATGAGTTAGAATAATA and seq. 3 AACGATGAATTACGTCGTAAA. Canine CLIP-170 target sequences: seq. a CACGCAGTTTGTGGAGTTAAA, seq. b AACTTCTATAATTGTATATAA, seq. c TAGAAAGTGTTTCACAAACAA and seq. d CAGGTGGAAGATGAAGCTAAT. Human IQGAP1 target sequences: seq. 1 CTGGGAGATAATGCCCACTTA, seq. 2 CAGGCGCTAGCTCATGAAGAA and seq 3 AATGCCATGGATGAGATTGGA (also targets canine sequence) [74]. For CLIP-170-rescue experiments, 2 µg of GFP-rat-CLIP-170 cDNA [59] was delivered using jetPRIME (Polyplus), according to the manufacturer's instructions.

### 4.5. CO-IP, cellular fractionation and SDS PAGE

Cell lysis and SDS PAGE were performed as described by James *et al.* [75]. For CO-IP experiments cells were lysed in M-PER mammalian protein extraction reagent (Pierce), mouse monoclonal CLIP-170 F-3 and mouse IgG (Sigma) were bound to Dynabeads protein G (Invitrogen) and Co-IP then performed as per the manufacturer's protocol. Rabbit polyclonal antibodies against β-actin (Abcam), α-tubulin and β-catenin were used at 1 : 10 000. Rabbit polyclonal antibodies against ninein (Bethyl), CLIP-170 and IQGAP1 H-103 were used at 1 : 2000. Mouse monoclonal antibodies against IQGAP1 and E-cadherin were used at 1 : 8000 and CLIP-170 F-3 at 1 : 2000. Secondary HRP antibodies produced in goat (Sigma) were used at 1 : 10 000. The membrane was analysed using a chemiluminescence detection kit (GE Healthcare). For re-probing, membranes were stripped in reblot solution (Chemicon) and antibody incubation and detection were repeated.

For cellular fractionation experiments, cells were lysed in fractionation buffer (250 mM sucrose, 20 mM HEPES pH 7.4, 10 mM KCl, 1.5 mM MgCl_2_, 1 mM EDTA, 1 mM EGTA, 1 mM DTT) for 20 min at 0°C, the nuclear and mitochondria fractions were fractionated and discarded by consecutive centrifugation at 720*g* and 10 000G. The remaining supernatant containing the membrane and cytosol fractions were separated by ultracentrifugation at 100 000*g* for 1 h (pellet contains membrane fraction). Each fraction was then analysed using SDS PAGE as above, with E-cadherin and α-tubulin identifying the membrane and cytosol fractions, respectively.

### 4.6. Microscopy and statistical analysis

Cells were imaged on a Zeiss Axiovert 200M microscope and a Zeiss LSM510 META confocal microscope. Images were processed using Axiovision (Zeiss) and Photoshop (Adobe) software.

Data for statistical analysis were first assessed for normal distribution using the D'Agostino and Pearson normality test. If the data were normally distributed, a parametric *t*-test or one-way ANOVA was applied. For datasets too small for normal distribution analysis and data not normally distributed, a non-parametric Mann–Whitney *U*-test or non-parametric Kruskal-Wallis test (with Dunn's multiple comparison post-test) was used to determine significance.

MT cell–cell cortical targeting was assessed in 10 × 10 µm cortical boxes with the percentage of MTs approaching the cortex at perpendicular angles (45–90°) calculated per box and then analysed. When junctional labelling was possible, the number of cortical contacts and MT orientation were measured and analysed. MTs making cortical contact were counted for every 10 µm of junction (using β-catenin staining). The ImageJ (FIJI) plugin ‘FibrilTool’ [42] was used for analysis of orientation of MTs to cell–cell junctions. This tool uses the circular average of gradients in pixel intensity across a given region of interest to find the predominant orientation and extent of alignment of ‘fibrillar structures’. It has previously been used in the quantification of the organization of plant cortical MTs [76] and was used here to measure the general orientation of MTs relative to junctions.

For organoid analysis, the number of budding events per organoid was counted on day 2, day 4 and day 6 following passaging into fresh Matrigel. This was performed in 10 regions from three independent experiments with more than 6000 organoids assessed. For each region, the percentage of organoids with no buds, 1 bud, 2 buds, 3 buds and 4 buds or more was calculated.

Whole cell EB1 and centrosomal ninein intensity were measured using Volocity software (Improvision) using fixed exposure images, and significance was assessed. Protein intensity analysis at cell junctions was performed using Andor iQ2 (Andor Technology). Fluorescence intensity line profiles through cell–cell junctions were measured from fixed exposure images with 21 readings taken over 2 µm. The data were averaged and base-lined against background intensity. For analysis of peak intensity at cell junctions, peak readings were normalized against control cells. Blind CLIP-170 comet analysis was performed in regions (10 × 10 µm boxes) on fixed exposure images with background subtraction, threshold and particle size (0.2–1.2 µm^2^) all applied equally to each image using ImageJ software; the number of comets per region were analysed.

GFP-CLIP-170 comet trajectories were obtained using uTrack, previously packaged as plusTipTracker [43]. All post-tracking analysis was conducted using MATLAB (MathWorks, 2013b) code written in-house (see electronic supplementary material for further details). To filter out tracks that were abnormally bendy, tracks were split where the orientations of consecutive segments differed above a threshold of 30°. To get better resolution at low to medium speeds (approx. 5–15 µm min^−1^) while comparing speeds of growth tracks between treatments, the mean speed plus one standard deviation from the ‘fastest’ cell (which was a control cell) was taken as the maximum speed in the analysis.

## Supplementary Material

Supplementary data

## Data accessibility

The datasets supporting this article have been uploaded as part of the electronic supplementary material.
