# Supplementary material for: Ninein is essential for apico-basal microtubule formation and CLIP-170 facilitates its redeployment to non-centrosomal microtubule organizing centres
Source: Open Biol. 2017 Feb 8;7(2):160274. doi: 10.1098/rsob.160274 (PMC5356440; doi:10.1098/rsob.160274)
Supplement: Supplementary data [file rsob160274supp1.pdf]

Supplementary data

Ninein is essential for apico-basal microtubule formation and CLIP-170 facilitates its redeployment to non-centrosomal Microtubule Organising Centres

Deborah A Goldspink, Chris Rookyard, Benjamin J Tyrrell, Jonathan Gadsby, James Perkins, Elizabeth K Lund, Niels Galjart, Paul Thomas, Tom Wileman and Mette M Mogensen

Open Biology DOI: 10.1098/rsob.20160049

Supplementary Movie 1: Control ARPE-19 GFP- CLIP-170 dynamics

Time-lapse of a control ARPE-19 cell transiently expressing GFP-CLIP-170. Images taken every 3 seconds over a 3 minute period (60 frames in total) and replayed at 5 frames per second. Second half of the movie shows U-Track analysis of GFP-CLIP-170 comets over this period with growing (blue), shrinking (pink) and pausing (yellow) MTs highlighted.

Supplementary Movie 2: Rac1-inhibited ARPE-19 GFP- CLIP-170 dynamics

Time-lapse of a Rac1-inhibited (12hrs 250μM NSC23766) ARPE-19 cell transiently expressing GFP-CLIP-170. Images taken every 3 seconds over a 3 minute period (60 frames in total) and replayed at 5 frames per second. Second half of the movie shows U-Track analysis of GFP-CLIP-170 comets over this period with growing (blue), shrinking (pink) and pausing (yellow) MTs highlighted.

Fig.S1

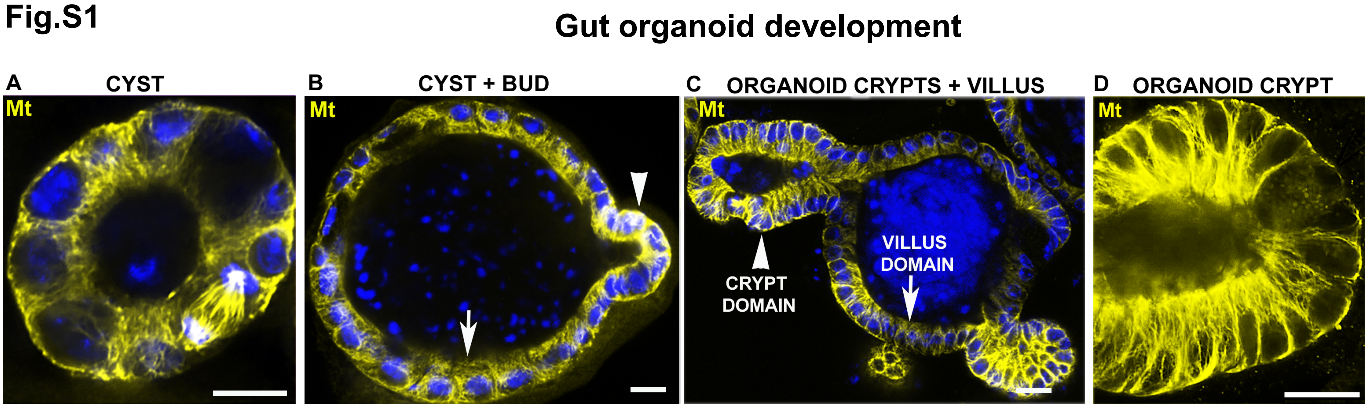

Fig.S1: Gut organoid development and microtubule organisation. A-C: Confocal images of stages of organoid development from early cyst (A) to bud (B arrowhead) and crypt (C) formation showing apico-basal microtubules (yellow), planar spindle alignment and nuclei stained with DAPI (blue). The stem cell region is located at the base of the crypts while fully differentiated villus-like cells are evident in older cyst (arows in B and C). **D:** Crypt including stem cell region at base showing epithelial cells with distinct apico-basal organised microtubules**.** Scale bars: 10μm

Fig.S2

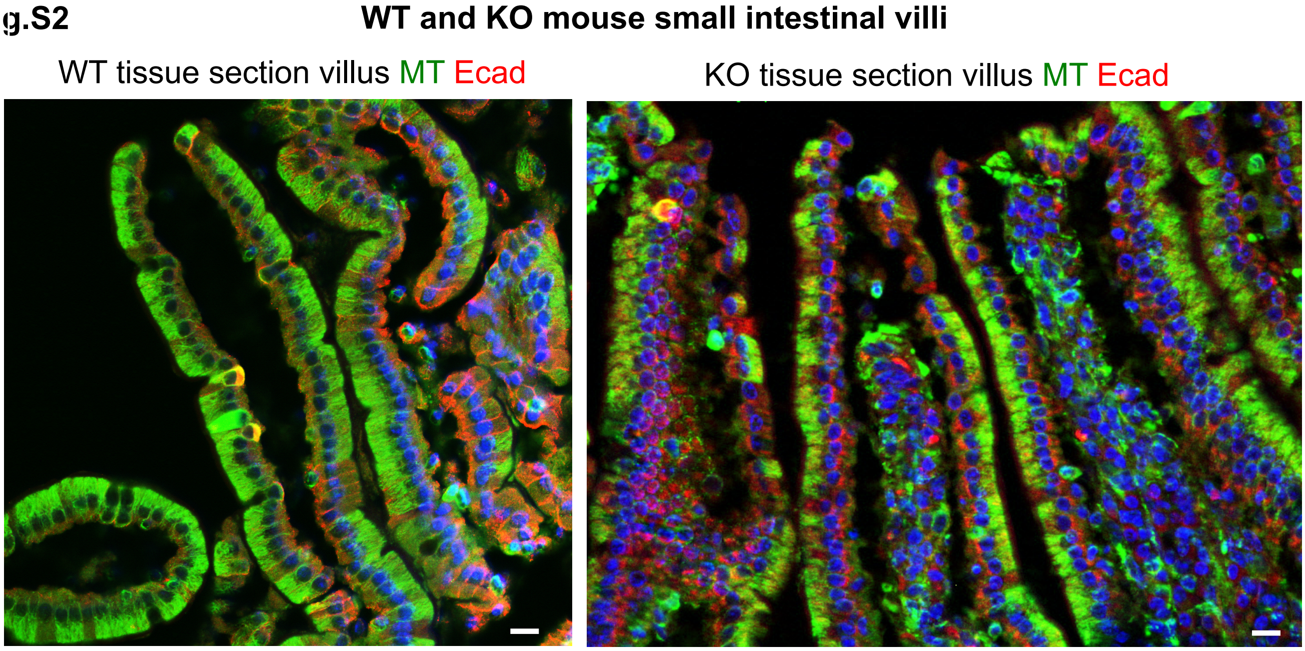

Fig.S2: Paraffin embedded sections of WT and KO small intestine labelled for MTs (green) and E-cadherin (red) showing villi with columnar cells with basal nuclei**.** Nuclei stained with DAPI (blue). Scale bars: 10μm

Fig.S3

Isolated WT and KO mouse small intestinal villi

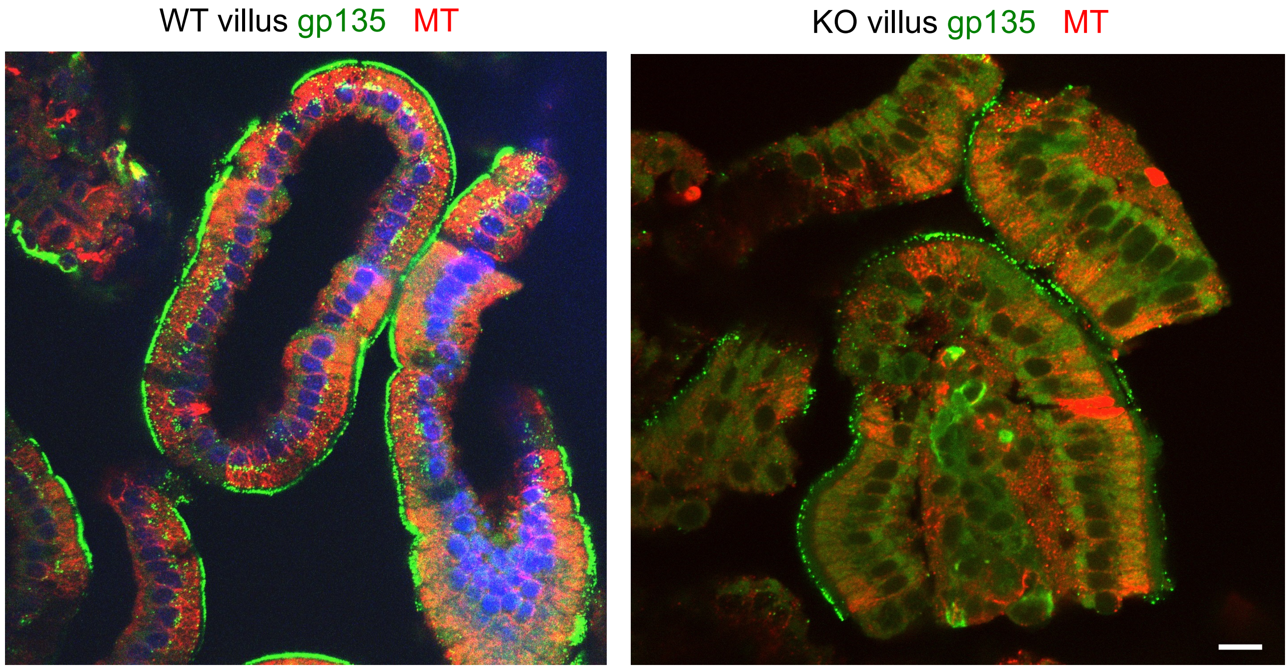

Fig.S3: Villi whole mounts from WT and *CLIP1/CLIP2* double KO small intestinal stained for the apical marker gp-135 (green), MTs (red) and DAPI (blue) indicating less apical gp135 localisation in KO compared to WT. Scale bar = 10µm

Fig.S4

Decreased acetylated tubulin in KO villus

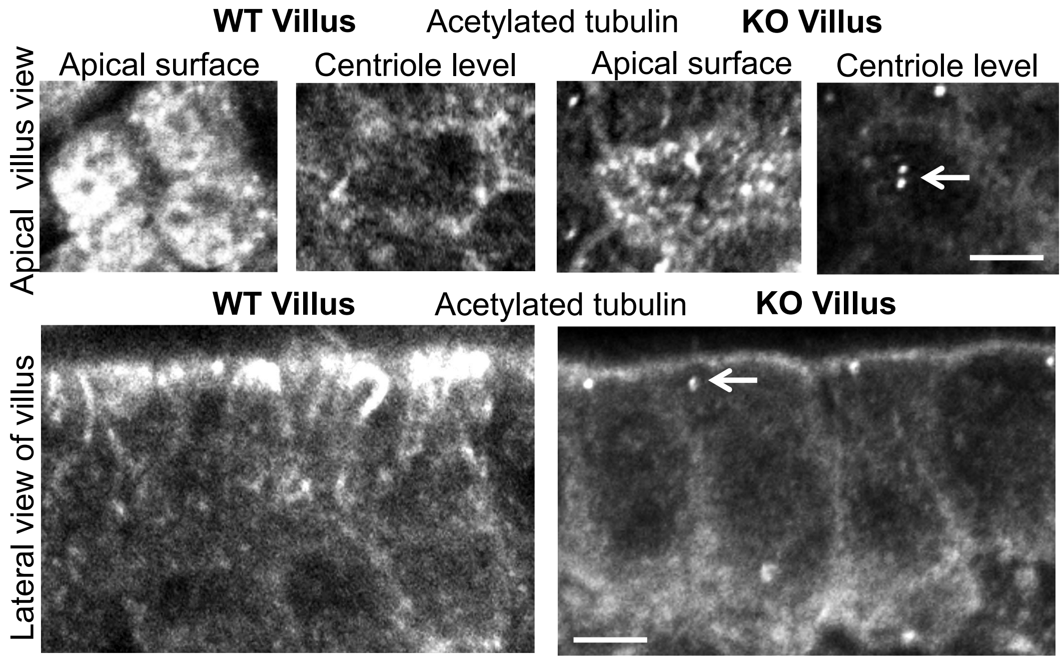

Fig.S4: Whole mount villus epithelium stained for acetylated tubulin, with apical cross-sections (apical views) and lateral views highlighting acetylation of apico-basal MTs in WT cells, but indicating little acetylation in KO cells. Note also that the KO cells contained apical centrioles (arrows), which were absent in WT cells. Scale bars = 5µm

Fig.S5

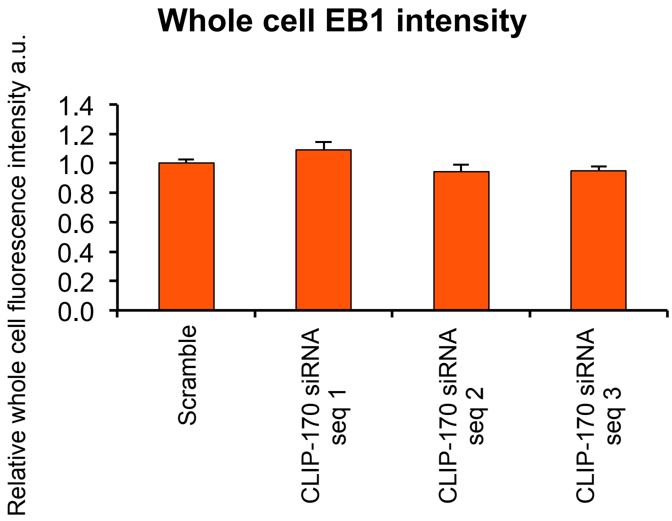

Fig.S5: Mean whole cell EB1 fluorescence intensity in ARPE-19 cells from mixed cultures of scramble and CLIP-170 siRNA (seq 1-3) showing no significant differences.

Fig.S6A **Rac1, IQGAP1 and ninein localisation in confluent MDCK**

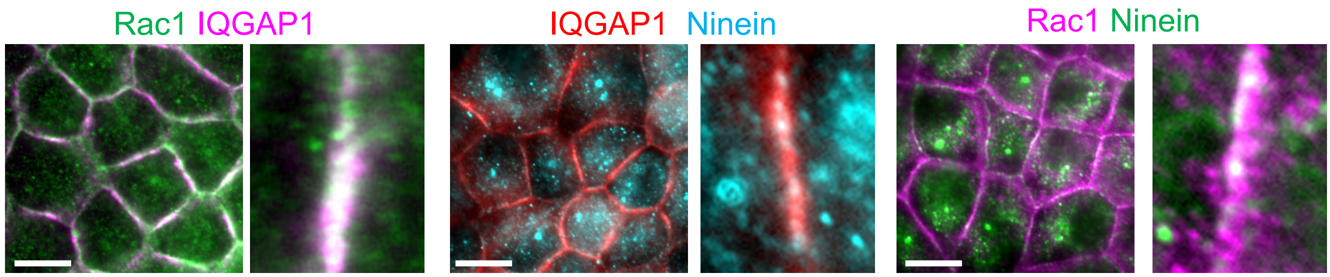

Fig.6A: Confluent MDCKII cells labelled for Rac1, IQGAP1 and ninein with enlarged regions highlighting cortical co-localisation. Scale bars = 10μm

Fig.S6B **Rac1 localisation in ARPE-19 cells**

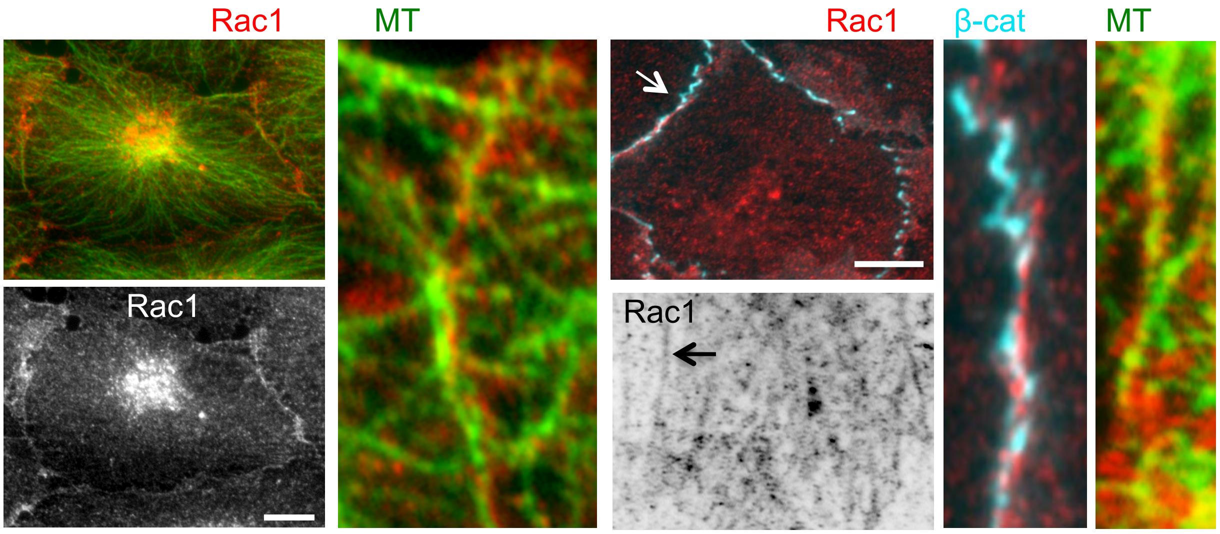

Fig.6B: Confluent ARPE-19 cells showing Rac1 (red) localisation together with either MTs (green) or β-catenin (blue). Enlarged regions highlight Rac1 accumulation at cortical sites (white arrow) and along MT filaments (black arrow). Scale bars = 10μm.

Fig. S6C: Effect of Rac1 inhibition with 250μM NSC23766 on actin organisation

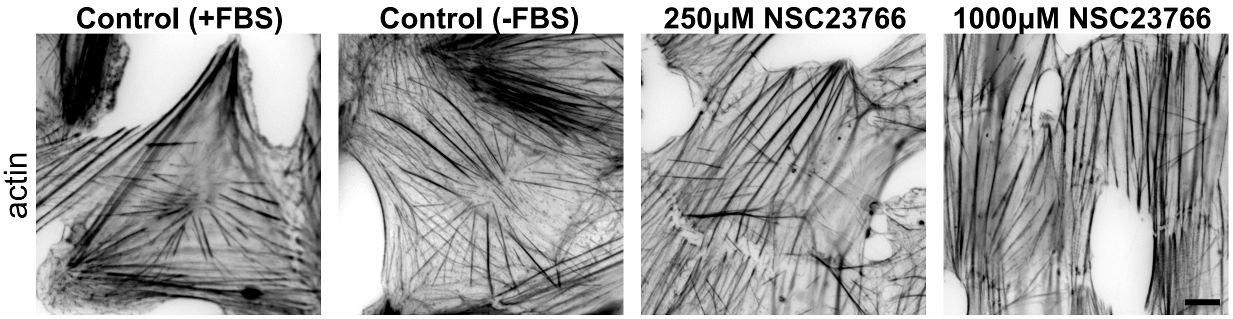

Fig.6C: Phallodin staining of control ARPE-19 cells grown with and without serum and Rac1-inhibited cells (NSC23766 250-1000μM). Note loss of dorsal actin fibres and increase in ventral stress fibres in response to Rac1 inhibition. Scale bars = 10μm.

Fig.S7 WT villus tissue reveal apical CAMSAP2 and cytoplasmic speckles

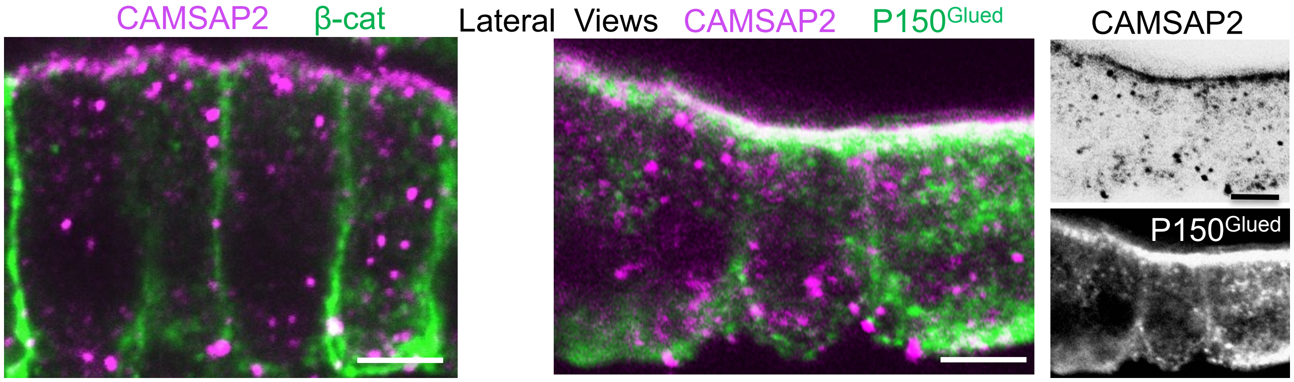

Fig.S7: Isolated WT villus tissues stained for CAMSAP2 (purple) and β-catenin (green) or $p150^{Glued}$ (green) showing a concentration of CAMSAP2 puncta at apical n-MTOCs with some co-localisation with $p150^{Glued}$.

Post-tracking microtubule dynamics analysis

The microtubule plus tip tracking package, “plusTipTracker”, takes as its input a series of TIFF files, one for each frame of a plus tip tracking film, and produces a number of output arrays that contain information about the dynamics of the microtubules – see Applegate et al. (2011) for a thorough discussion of the methodology and implementation of this package – A brief summarise of the plusTipTracker data that we used for our analysis is given below.

The main output from plusTipTracker is a list of “sub-tracks”, which are simply episodes of growth, shrinkage, or pausing that have been detected by the software. Usually, these sub-tracks will be part of a series of sub-tracks that have been linked together, i.e. one set of sub-tracks can include episodes of all microtubule phases, and these linked tracks are called “compound tracks”. In addition to the list of sub-tracks, there are also two matrices containing the x- and y-coordinates for the linked sub-tracks. These coordinate matrices are n-by-f in size, where n is the number of compound tracks, and f is the number of frames in the film; thus each compound track has a row in the coordinate matrices and each frame has a column. It is possible to cross-reference between the sub-track list and the coordinate matrices; the sub-track list has entries for the compound track to which it belongs (i.e. the row in the coordinate matrices), and for the start and end frames of its detection (i.e. the start and end columns in the coordinate matrices).

We used the list of sub-tracks along with the coordinate matrices in our analysis. The sub-track list also contains entries for the type of track it is, along with its length and speed. Thus, we could calculate the percentages of tracks in a given phase, and the average growth speeds and lengths. We removed overly circuitous growth tracks from our analysis. From the coordinate matrices, we could calculate the orientation of a track between frames, and when two consecutive track orientations differed by more than 30°, we split the track at that point. After splitting circuitous tracks, average track times, velocities and lengths were recalculated. For the comparisons of growth speeds between control and Rac1-inhibited cells, we wanted to impose an upper limit on growth speeds so that we would be able to better compare low to medium speeds. For this upper limit, we looked at the average growth speeds per cell, and found the maximum (a control cell). We then added the standard deviation of speeds from that cell to the average, and used that as our upper limit. We then split growth speed values into four evenly sized groups for each treatment.

The Matlab code for the analyses described was written on an ad hoc basis and thus is not expected to be of immediate use to any general analysis. However, the code is available from C.R. upon request.
Email:christopher.rookyard@kcl.ac.uk
